# vProtein: Identifying Optimal Amino Acid Complements from Plant-Based Foods

**DOI:** 10.1371/journal.pone.0018836

**Published:** 2011-04-22

**Authors:** Peter J. Woolf, Leeann L. Fu, Avik Basu

**Affiliations:** Foodwiki, Ann Arbor, Michigan, United States of America; University College Dublin, Ireland

## Abstract

**Background:**

Indispensible amino acids (IAAs) are used by the body in different proportions. Most animal-based foods provide these IAAs in roughly the needed proportions, but many plant-based foods provide different proportions of IAAs. To explore how these plant-based foods can be better used in human nutrition, we have created the computational tool vProtein to identify optimal food complements to satisfy human protein needs.

**Methods:**

vProtein uses 1251 plant-based foods listed in the United States Department of Agriculture standard release 22 database to determine the quantity of each food or pair of foods required to satisfy human IAA needs as determined by the 2005 daily recommended intake. The quantity of food in a pair is found using a linear programming approach that minimizes total calories, total excess IAAs, or the total weight of the combination.

**Results:**

For single foods, vProtein identifies foods with particularly balanced IAA patterns such as wheat germ, quinoa, and cauliflower. vProtein also identifies foods with particularly unbalanced IAA patterns such as macadamia nuts, degermed corn products, and wakame seaweed. Although less useful alone, some unbalanced foods provide unusually good complements, such as Brazil nuts to legumes.

Interestingly, vProtein finds no statistically significant bias toward grain/legume pairings for protein complementation. These analyses suggest that pairings of plant-based foods should be based on the individual foods themselves instead of based on broader food group-food group pairings. Overall, the most efficient pairings include sweet corn/tomatoes, apple/coconut, and sweet corn/cherry. The top pairings also highlight the utility of less common protein sources such as the seaweeds laver and spirulina, pumpkin leaves, and lambsquarters. From a public health perspective, many of the food pairings represent novel, low cost food sources to combat malnutrition. Full analysis results are available online at http://www.foodwiki.com/vprotein.

## Introduction

The human body requires a small set of indispensible amino acids (IAAs) in a defined proportion. These IAAs are provided in roughly the same proportion in most animal-based foods, but are often found in different proportions in plant-based foods [1]. Humans have overcome imbalances in plant-based foods by consuming foods with complementary IAA patterns. Historic examples of these complements include beans and corn in the Americas [2], or rice and soy in Asia [3], [4]. However, given changes in food availability and an increase in data about food, what other plant-based food pairings could serve our needs as well or better than these historical complements? In this work we have developed a quantitative tool called vProtein to explore this question.

**Figure 1:**
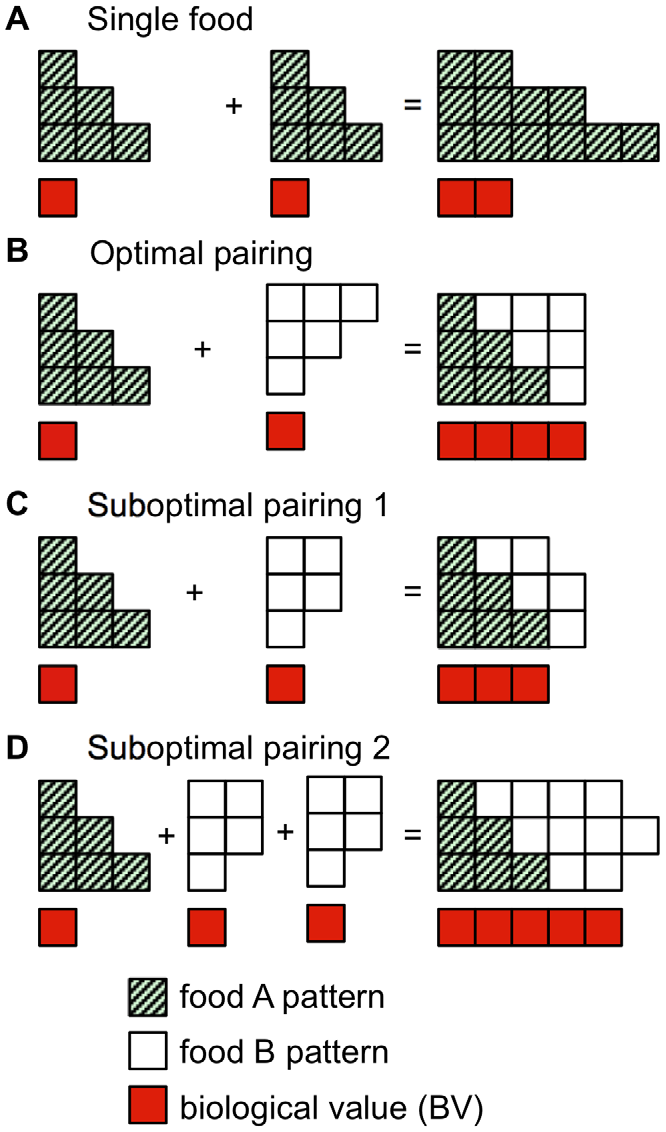
A simplifed example of complementation with three hypothetical amino acids (vertical axis). Note that the optimial pairing (B) is balanced in that all of the amino acids contribute to the biological value, while the suboptimal pairings (A,C, and D) are unbalanced in that there are excess amino acids (6, 2, and 1 units respectively) that do not contribute to the biological value.

Broadly, complementation involves consuming two or more foods together to yield an amino acid pattern that is better than the sum of the two foods alone. A simplified example of complementation with three hypothetical amino acids is shown in Figure 1. In this example, the number of units that contain a complete set of amino acids determines the biological value (BV) of the complement. Once one or more amino acids are depleted, protein synthesis cannot proceed. For a single food (Fig. 1A), there is no complementation, so doubling the food intake will yield double the BV. In contrast, pairing of two foods that are optimal complements (Fig. 1B) produces a synergistic effect where the two components alone yield 2 units BV, but together they yield 4 units BV. In the case shown in Figure 1B, a 1∶1 complement is optimal in the sense that there are no excess amino acids–thus all components of the food can be used with full 100% efficiency. If the pairing is suboptimal but still complementary (Fig. 1C,D), consuming the two foods together yields more biological value than each food alone, but leaves a varying quantity of amino acids in excess, resulting in less efficient combinations. As shown in Fig. 1D, pairing food A to food B in a 1∶2 ratio yields a more efficient pairing than the 1∶1 pairing in Fig. 1C. Thus, for any set of foods there is a particular ratio that will minimize the excess amino acids to produce the most efficient combination. An example of an optimized rice-soy complement is shown in Figure S1.

The relative proportion, or pattern, of IAAs required for human health has been the topic of considerable research. IAA patterns commonly discussed include the MIT pattern [5], Millward Pattern [6], the 1985 FAO/WHO/UNU pattern [7], and the 2005 dietary reference intake (DRI) pattern published by the Food and Nutrition Board [8]. These patterns are broadly similar to each other, and are similar to the IAA patterns observed in common animal-based protein sources such as chicken breast, egg, and milk as is shown in Figure S1.

A common use for IAA patterns is for calculating the protein digestibility corrected amino acid score (PDCAAS) for particular foods [9]. PDCAAS values range from 1.0 to 0, with 1.0 representing protein sources with high BV such as egg and milk. Although PDCAAS values are widely accepted, the method has two practical limitations as noted elsewhere [10], [11]. First, PDCAAS values do not indicate possible complements. Thus combining two foods with low PDCAAS values (low BV) may or may not yield a superior IAA pattern. As a result, using PDCAAS alone would overlook potentially important food complements that may be of high BV. Second, to calculate a PDCAAS value requires knowing the true fecal digestibility of the food. Unfortunately, the fecal digestibility has been measured for only a small set of foods, and it depends on how the food is processed. However in the foods measured the range of fecal digestibility is relatively small. For example, according to a 1990 FAO/WHO study, fecal digestibility ranged from 0.98 for egg to 0.91 for wheat, where 1.0 represents full digestibility [9].

As an alternative to using PDCAAS, we have developed an analytical tool called vProtein that uses only IAA patterns to evaluate combinations of foods. Using IAA patterns provided by the United States Department of Agriculture standard release 22 (USDA sr22) database, we use vProtein to identify single foods and pairs of foods that yield an IAA pattern most similar to the 2005 dietary reference intake (DRI) pattern [8]. vProtein identifies the optimal weighting of each food using a linear programming approach. A similar approach has been successfully used in earlier work to estimate the ingredient fraction in processed foods [12]. The results identify both traditional and unexpected couplings and, in so doing, provide a data-driven resource to help inform dietary decisions.

## Results

### Plant-based food library

A total of 1251 plant-based foods were identified for use in the subsequent analysis. These foods fell into the following USDA defined food groups: Vegetables and Vegetable Products (559); Legumes and Legume Products (186); Cereal Grains and Pasta (153); Fruits and Fruit Juices (131); Nut and Seed Products (125); Breakfast Cereals (68); Spices and Herbs (19); Fats and Oils (6); and Beverages (4).

### Single food analysis

Each single food was analyzed to determine the mass of the food required to obtain an equivalent of 1 gram of high BV protein as defined by the 2005 DRI pattern. Of the 1251 foods analyzed, some single foods stood out as unusually balanced or unbalanced based on the mass of excess IAAs. A summary of the most balanced and unbalanced foods is provided in Tables 1 and 2 and described in detail on the vProtein website (http://www.foodwiki.com/vprotein). IAA patterns from a selection of the unbalanced foods are provided in Figure S3.

**Table 1:** Top single foods with the most balanced IAA patterns resulting in the highest IAA efficiencies.

Wheat based formulated nuts
Wheat germ
Quinoa
Pickle Relish
Cauliflower
Garlic
Cinnamon
Hummus
Tomatoes
Acorns

**Table 2:** Top single foods with the most unbalanced IAA pattern resulting in the lowest IAA efficiencies.

Food	IAAs most in excess (+) or deficient (−)
Macadamia nuts	(−) lysine; (−) methionine+cysteine
Corn based breakfast cereals	(−) lysine; (+) leucine
Degermed cornmeal	(−) lysine; (+) leucine
Wakame seaweed	(+) valine; (−) histidine; (−) lysine
Peeled cucumber	(+) tryptophan; (−) histidine
Prepared mustard	(−) tryptophan
Peas with edible pods	(+) valine; (−) histidine
Apricots	(+) tryptophan; (−) methionine+cysteine
Cranberries	(−) tryptophan; (−) methionine+cysteine
Millet	(−) lysine
Brazil nuts	(+) methionine+cysteine

We note that the most unbalanced foods are almost always markedly deficient or in excess in a single IAA. Although the most commonly deficient IAAs are lysine and the sulfur-containing IAAs of methionine and cysteine, there are some unusual exceptions. For example, prepared mustard is nearly devoid of tryptophan, while peeled cucumber is exceptionally low in histidine.

### Food pair analysis

The best overall food pairings observed in this analysis are shown in Table 3. The top pairings are divided between pairings that minimize calories, minimize weight, and maximize efficiency. In general, there is significant overlap in pairings that minimize calories and weight—both of which show a dominant pattern of pairing soy products with a complementary protein rich food.

**Table 3:** Ranked top food pairings based on combinations that minimize total calories, maximize IAA efficiency, and minimize total weight.

Optimization Goal	Food 1	Food 2
Minimize calories	Sesame seed flour	Soy protein isolate
	Seaweed, spirulina	Soy protein isolate
	Seaweed, laver	Soy protein isolate
	–	Soy protein isolate
	Cottonseed flour	Soy protein isolate
	Sunflower seed flour	Soy protein isolate
	Seaweed, spirulina	Soy sauce (tamari)
	Seaweed, spirulina	Watercress, raw
	Seaweed, spirulina	**
	Seaweed, spirulina	Pumpkin leaves
--------------------	--------------------	--------------------
Maximize efficiency	Sweet corn, whole kernel	Tomatoes
	Apple	Coconut meat
	Sweet corn, whole kernel	Sweet cherries
	Orange or tangerine juice	Edamame
	Lambsquarters	Barley malt flour
	Sweet corn, whole kernel	Wheat germ
	Dates, Medjool	Edamame
	Sweet corn, whole kernel	Lotus root
	Apple	Mustard seed
	Sweet corn, whole kernel	Peppers, sweet
--------------------	--------------------	--------------------
Minimize weight	Sesame seed flour	Soy protein isolate
	**	Soy protein isolate
	Cottonseed flour	Soy protein isolate
	Sunflower seed flour	Soy protein isolate
	Brazil nuts	Soy protein isolate
	Seaweed, spirulina	Soy protein isolate
	Watermelon seed kernels	Soy protein isolate
	Safflower seed flour	Soy protein isolate
	Butternuts	Soy protein isolate
	Peanut flour	Soy protein isolate

In some cases, single foods (denoted by a ** in the alternate entry) outperformed food pairs. Note these tables are summaries of the larger list on the vProtein website (http://www.foodwiki.com/vprotein).

The top food pairs that maximize the efficiency of IAA usage (Table 3) contain a wider diversity of foods. These pairs represent the top pairings exclusively from an IAA viewpoint, and include foods that are not generally viewed as protein sources such as apples and orange juice.

### Food group pairings

Examining the top 100 pairings for each food, we found no consistent pattern of food group-food group pairings. This observation was confirmed using a chi-squared test and indicated that no food group pairing was overrepresented compared to a random sampling at a p-value of 0.05 or less. Details of the top pairings by food group and chi-square statistics are provided in Tables S1, S2, and S3.

### Variability in amino acid compositions

The calculated pairings made by the vProtein algorithm assume that the IAA composition of each food is accurately known and unchanging. However, the nutrient composition of any food is likely to vary depending on the variety, storage and preparation conditions, and growth environment. To assess the variability of each IAA, we examined the standard error of the mean for each IAA measurement on a plant-based food in the USDA sr22 database. Expressed as a percent error, we found the following average error values: histidine 3.3%; tryptophan 2.7%; threonine 3.8%; isoleucine 2.5%; leucine 3.6%; lysine 3.3%; methionine 4.2%; cystine 4.4%; phenylalanine 2.9%; tyrosine 3.4%; valine 1.9%.

To assess the impact of this level of variability, we resampled all of the IAA data using a worst case of 5% error and reran the vProtein analysis. The resulting two-way pairings were nearly identical with only slight changes in ranking and weight calculations for each component (data not shown). This result indicates that although the IAA content of these foods does vary, this variability does not significantly affect the predictions made by the analysis.

## Discussion

Our analyses identified a large number of single and pairs of plant-based foods that satisfy the 2005 DRI IAA pattern. Some of these foods represent historically well-known protein sources such as soy products. In addition, the analysis has uncovered a number of less well-known protein sources, such as the pairings listed in Table 3. In some cases, these less well-known protein sources are not protein dense foods, but are eaten in large enough quantities to contribute significant protein to a diet.

Note that in the current analysis, vProtein only identifies food pairs based on the measured IAA profile in the food, and as such does not account for the other macronutrient needs a person may have.

### Food group pairings

Before starting this analysis, we expected historically well-known pairings such as grain/legume to dominate the pairings. In contrast, no bias was observed in the list of top pairings when analyzed by food group. The lack of a statistically significant food group-food group association could be explained in two ways. First, it is possible that the food group labels assigned by the USDA are introducing artifacts into the analysis. For example, dried soybeans are listed as “Legumes and Legume Products,” while fresh soybeans (edamame) are listed as “Vegetables and Vegetable Products.” However, manual inspection of the food pairings reveals that nearly all foods are matched to a wide diversity of foods seemingly independent of possible food group labeling errors.

A second possible reason why no significant food group-food group pairings were found is that food groups are poor predictors of IAA patterns. Traditional protein pairings of legumes and grains are based on the assumption that legumes are generally limited in methionine or cysteine, while grains are limited in lysine [1]. While this observation is often true, each food has a somewhat different IAAs pattern that has better and worse complements. Furthermore, there are apparently other foods that provide at least as good if not better complements that are in different food groups. For example, when minimizing excess vProtein finds “Wheat flour, whole-grain” (USDA20080) is complemented by split peas or chickpeas (grain/legume combinations), but the flour is also well complemented by lambsquarters or raw cauliflower (grain/vegetable combinations), and apples (a grain/fruit combination).

These analyses suggest that pairings of plant-based foods should be based on the individual foods themselves instead of based on a broader food group/food group pairing. By analyzing foods on a case-by-case basis, one can also define the proportion of each food required for completeness. In this analysis, food pairings ranged from less than 1% weight of one food to nearly balanced proportions—depending on the food and the optimization objective.

### Applicability of pairings to processed complementary foods (PCF)

PCF have been widely explored as a method for supplementing infant and child food sources in resource poor areas. PCFs have the advantage of storability and the ability to prepare these meals one serving at a time [13]. Empirically, PCFs have been successfully used to improve growth and macronutrient status in a number of environments [14], [15], [16].

The basis of most PCFs is a micronutrient-fortified mixture of soy and rice. In this context, soy and rice provide low cost and storable sources of both calories and protein. When analyzed in vProtein, the top weight minimizing complements for “Rice flour, white” include “Cereals ready-to-eat, wheat germ, toasted, plain” (95%) followed by a long list of soy protein products. Optimal pairings of rice to soy protein products indicate an optimal ratio from 60-25% rice, depending on the soy product. This pairing result is in agreement with the observed impact of PCFs on improving growth, supporting the validity of the vProtein analysis results.

Interestingly, when the vProtein analysis is repeated to find complements to “Soy flour, defatted” we find that rice does not score well as an optimal weight minimizing complement. Instead the top scoring pairings include dried spirulina (30%), variants of cottonseed flour (15%), various forms of sesame flour (12-8%), brazil nuts (12%), safflower seed meal (17%), watermelon seed (15%), and defatted peanut flour (28%). Interestingly, in the top 100 pairings, rice only appears as rice bran.

The soy pairings identified by vProtein have two possible advantages over the rice flour pairings. First, the soy pairings produce more weight efficient combinations. For example the top scoring rice flour combinations have a minimum total weight of 73 grams to provide 25 grams of high quality protein, while the soy combinations have a minimum total weight of 45 grams to provide 25 grams of high quality protein. The additional weight of the rice complements is mainly due to starch, which may or may not be desirable as a calorie source. Overall the top rice complements provide from 260 to 360 calories, while complements to “Soybeans, mature seeds, raw” provide 180 to 260 calories.

The second advantage of the soy pairings is that they encompass a more diverse space of possible foods. This diverse space provides both flexibility in food sourcing along with a greater potential diversity of other nutrients. As an example, pairing soy to spirulina also introduces a wide variety of micronutrients, thereby reducing the amount and potentially cost of micronutrient supplementation. Field trials with spirulina supplementation to a soy, millet, and peanut mixture have shown a synergistic relationship in rehabilitating undernourished children [17]. From a public health perspective, these alternative food pairings provide examples of viable protein sources that could be locally produced in a wide variety of climates.

### Higher order combinations

We expect that three food and higher order combinations will be able to match the reference IAA pattern with rapidly increasing accuracy. Ideally, the net IAA pattern from a complete meal would be examined because this is the IAA combination that the body experiences. However, exhaustively searching even all 3 way complements of the 1251 plant based foods discussed in this paper yields on the order of 1 billion possible combinations. This large search space is both computationally prohibitive and the resulting combinations are difficult to visualize.

### Unbalanced foods as complements

The foods with the most unbalanced IAA patterns listed in Table 2 represent an interesting list of possible complements, or even functional foods, due to their relatively rich or poor IAA pattern relative to our needs. For example, foods that are particularly enriched in a single IAA could be viewed as functional in that the food provides an unusually good complement to other foods, or the enrichment of the IAA itself may have physiological functions.

As an example of a possible functional food, the foods based on degermed corn contain a large excess of leucine relative to our needs (Figure S3B). These degermed corn based foods include cornmeal, corn based breakfast cereals, masa, and hominy. Research by others has suggested that leucine regulates cell proliferation via the mTOR pathway [18], [19], [20]. Furthermore leucine supplementation studies have suggested that leucine acts as an endogenous indicator of amino acid status [21], [22] and is partially responsible for skeletal muscle maintenance [22], [23], [24]. Given these observations, the excess of leucine in degermed corn products could provide a physiologically relevant imbalance of leucine in some diets.

The IAA imbalance in Brazil nuts is also nutritionally interesting because Brazil nuts only contain a significant excess of methionine and cysteine (Figure S3E). This particular imbalance is of interest because many legumes are limited by methionine and cysteine. This relative abundance of sulfur containing amino acids in the 2S albumin fraction of Brazil nuts has been identified as a possible transgenic improvement to soy to complement its amino acid efficiency [25]. The complementarity of Brazil nuts to legumes is clearly shown in vProtein. For example, to obtain 25 grams of high BV protein requires 492 grams of canned pinto beans (USDA16044) for a total calorie intake of 423 kcal. When paired with 12 g of Brazil nuts (USDA12078), we require only 364 g of canned pinto beans, for a total of 391 kcal. This small addition of Brazil nuts yields a 23% reduction in the total food mass and a 7.5% reduction in calories. In this case, Brazil nuts could be viewed as a functional food in that only a small amount of the nut is needed to complement the IAA pattern of legumes particularly well.

Using a quantitative, informatics based approach we are able to systematically explore areas of food space to optimize nutrient patterns in general, and not just for IAA patterns. A similar approach could be used to identify food combinations that contain desirable lipid, carbohydrate, mineral, and vitamin patterns, for example. These quantitative diets may or may not map to historical diets, but would better reflect our physiological needs based on current research.

## Methods

### Nutrient data preprocessing

All analyses were based on a set of IAA measurements for plant-based foods. The IAA measurements were obtained from the U.S. Department of Agriculture National Nutrient Database for Standard Reference, Release 22 (USDA sr22) [26]. Within this database, we selected only foods in the database that have IAA measurements.

Next, from the set of foods with IAA measurements, we identified plant-based foods using the following two steps. First, foods were limited to the following USDA food groups: Vegetables and Vegetable Products; Legumes and Legume Products; Cereal Grains and Pasta; Fruits and Fruit Juices; Nut and Seed Products; Breakfast Cereals; Spices and Herbs; Fats and Oils; and Beverages. We recognize that other plant-based foods exist in the database, such as in the USDA food groups Ethnic Foods, Snacks, and Fast Foods, however upon further inspection we found that few if any of these entries contained IAA measurements. Second, we manually eliminated any animal-based foods that were suspected to contain dairy, eggs, or honey.

### IAA reference pattern

As an IAA reference pattern, vProtein uses the pattern in the 2005 dietary reference intake (DRI) published by the Food and Nutrition Board [8]. The DRI pattern was selected because it is one of the best-accepted IAA patterns available. The DRI reference pattern is based on the estimated average requirements for a 1- to 3-year-old human and has been adopted as the US national reference value. This pattern is as follows (in mg/g of protein): isoleucine, 25; leucine, 55; lysine, 51, methionine+cysteine (SAA), 25; phenylalanine+tyrosine, 47; threonine, 27; tryptophan, 7; valine, 32; and histidine, 18 [8].

A comparison of the 2005 DRI pattern, other commonly used reference patterns, and the IAA patterns in foods commonly identified as complete proteins (egg white, whey protein, milk, and chicken breast), demonstrates the similarity between these patterns (Fig. S2). This similarity suggests that changes to a different reference IAA pattern would have only a small change on the analyses presented in this work. Indeed, when the reference pattern was changed to raw egg white, we observed largely similar results in terms of predicted food pairings (data not shown).

Note that the IAA pattern used in this work is a generally accepted consensus profile, but may not optimally apply to all age groups, environmental conditions, or life stage. As an example, work in 2010 suggests a slightly different IAA pattern for 2 year old children [27]. Optimal plant based pairings based on alternative IAA patterns can be generated by rerunning the optimization described in this paper.

### Single food protein equivalent

For a single food, there exists a unique quantity that will provide at least the reference pattern of each IAA. This food quantity is determined by the most deficient IAA in the food—a similar process as is used to calculate the amino acid component of the PCDAAS value. Once the limiting IAA is found, the food quantity is rescaled by the reciprocal of the ratio of this IAA to the reference. This rescaling ensures that the food mass will contain exactly the reference value for the limiting IAA, and an excess of the remaining IAAs. Mathematically, this scaling weight can be expressed as:
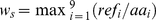
(1)Where *w_s_* is the scaled mass of the single food, *ref_i_* is the reference pattern value for IAA *i*, and *aa_i_* is the density of IAA *i* in the food.

### Food pair complement optimization procedure

Identifying pairs of complementary foods is more complex and requires two types of constraints. The first type of constraint requires that the food mixture contains at least the IAA content defined by the reference pattern. Alone, this constraint reduces the solution space, but does not define a unique weight for each food in the combination. To identify a single solution, we used a second constraint to minimize the total calories, total weight, or maximize efficiency by minimizing the total mass of IAAs beyond the reference pattern. Using these two kinds of constraints generates a standard linear programming problem.

Mathematically, these two groups of constraints can be expressed as the following:

Minimize one of the following:
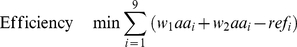
(2)

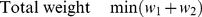
(3)

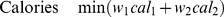
(4)Subject to:
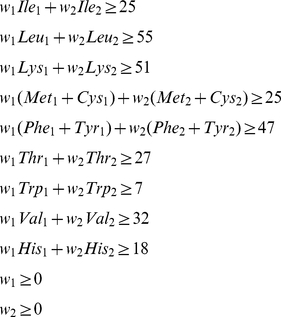
(5)Where *w_1_* and *w_2_* are the weights of foods 1 and 2 respectively, and the three letter codes define density of the IAA (mg amino acid per gram of food) in foods 1 and 2 as defined by the USDA database. Reference values on the right are from the 2005 DRI pattern discussed above. A similar linear programming framework was used in 1994 to correctly identify the ingredient composition of processed foods based on nutritional labeling data [12].

Note that by minimizing the total weight of the combinations, we bias the optimization toward combinations that are more practical from an eating perspective as they will tend to be smaller. By minimizing the total calories, we bias the search toward combinations with a higher protein to calorie ratio. By maximizing the IAA efficiency, we simultaneously minimize the mass of excess IAAs and minimize the total protein content of the combination while still satisfying the reference IAA pattern.

Optimization was carried out using the convex optimization package CVXOPT version 1.1.2 [28]. The optimization used a linear cone programming approach [29] to identify optimal combinations of foods that satisfy the requirements. If no optimal solution was found or if the resulting optimal combination included foods in excess of a 1000∶1 ratio, then the combination was dropped.

The relative weighting of all possible food pairings were tested, producing approximately 7.8×10^5^ pairs for each optimization condition.

### Statistical analysis of food group associations

High scoring food complements were analyzed to see if they mapped to a preferred USDA food group pairing. For example, were foods from the food group “Legumes and Legume Products” more frequently paired with foods from the group “Cereal Grains and Pasta?” The analysis was done separately for pairings identified from minimizing excess, calories, and weight. The list of observed pairs was made up of the top 100 pairs for each food, and then mapped to the food group for each food in the pairing. The resulting food group pairing frequency was then compared to the expected frequency of sampling the pair at random from our list of plant-based foods. This comparison was carried out using a Pearson's chi-square test to obtain p-values for each pair of foods.

### Data display

The analysis results were formatted for search and display online at the website http://www.foodwiki.com/vprotein. The interface was written in the python package web2py [30].

## Supporting Information

Figure S1 **A comparison of the essential amino acid reference patterns to chicken, whey, egg, and milk amino acid patterns.** All patterns are normalized for comparison, and as such only reflect the relative contributions from each essential amino acid but do not reflect the absolute scale. Note that the FAO/WHO/UNU, Millward, and MIT patterns do not include histidine. vProtein uses the 2005 DRI pattern.
(TIFF)

Figure S2 **An example pairing of tofu and brown rice.** (A) IAA profile of tofu and the corresponding one-way optimized result to obtain 25 grams of high quality protein. (B) IAA profile of brown rice and the corresponding one-way optimized result to obtain 25 grams of high quality protein. (C) IAA profile of the optimized combination of tofu and brown rice to obtain 25 grams of high quality protein. Note that the optimization result in C minimized the excess IAA concentration resulting in a maximally efficient IAA usage. This combination optimized for IAA efficiency is less weight efficient and less calorie efficient than tofu alone (386 g vs 237 g, and 398 kcal vs 216 kcal).
(TIFF)

Figure S3 A sampling of amino acid profiles of some of the particularly unbalanced foods listed in Table 2.
(TIFF)

Table S1 Minimum Excess: Overall food group based pairings, chi-square statistics, and top-food matches.
(DOCX)

Table S2 Minimum Calories: Overall food group based pairings, chi-square statistics, and top-food matches.
(DOCX)

Table S3 Minimum Weight: Overall food group based pairings, chi-square statistics, and top-food matches.
(DOCX)
