# Supplementary figures and images for: vProtein: Identifying Optimal Amino Acid Complements from Plant-Based Foods
Source: PLoS One. 2011 Apr 22;6(4):e18836. doi: 10.1371/journal.pone.0018836 (PMC3081312; doi:10.1371/journal.pone.0018836)

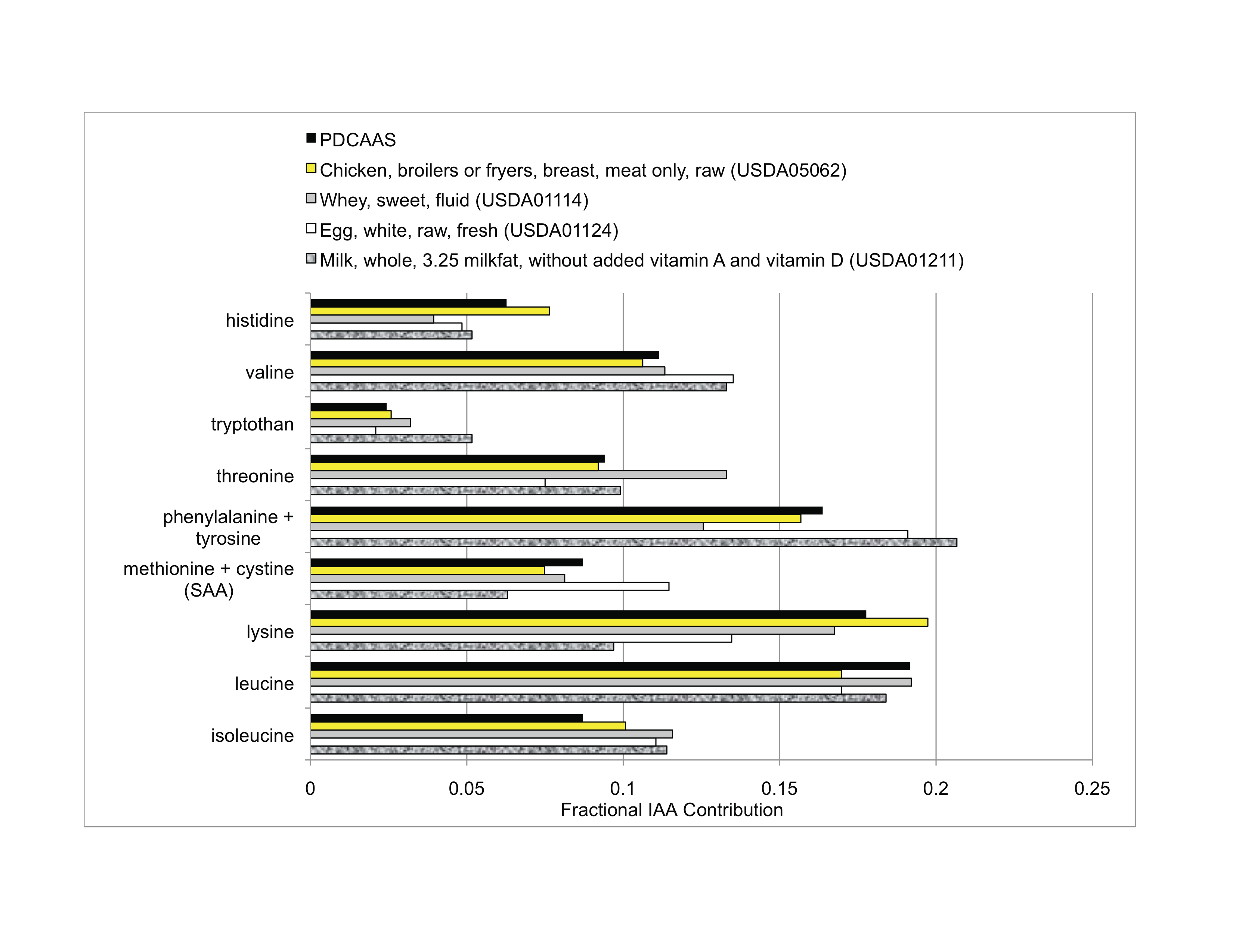

Supplement: Figure S1 — A comparison of the essential amino acid reference patterns to chicken, whey, egg, and milk amino acid patterns. All patterns are normalized for comparison, and as such only reflect the relative contributions from each essential amino acid but do not reflect the absolute scale. Note that the FAO/WHO/UNU, Millward, and MIT patterns do not include histidine. vProtein uses the 2005 DRI pattern. (TIFF) [file pone.0018836.s001.tiff]

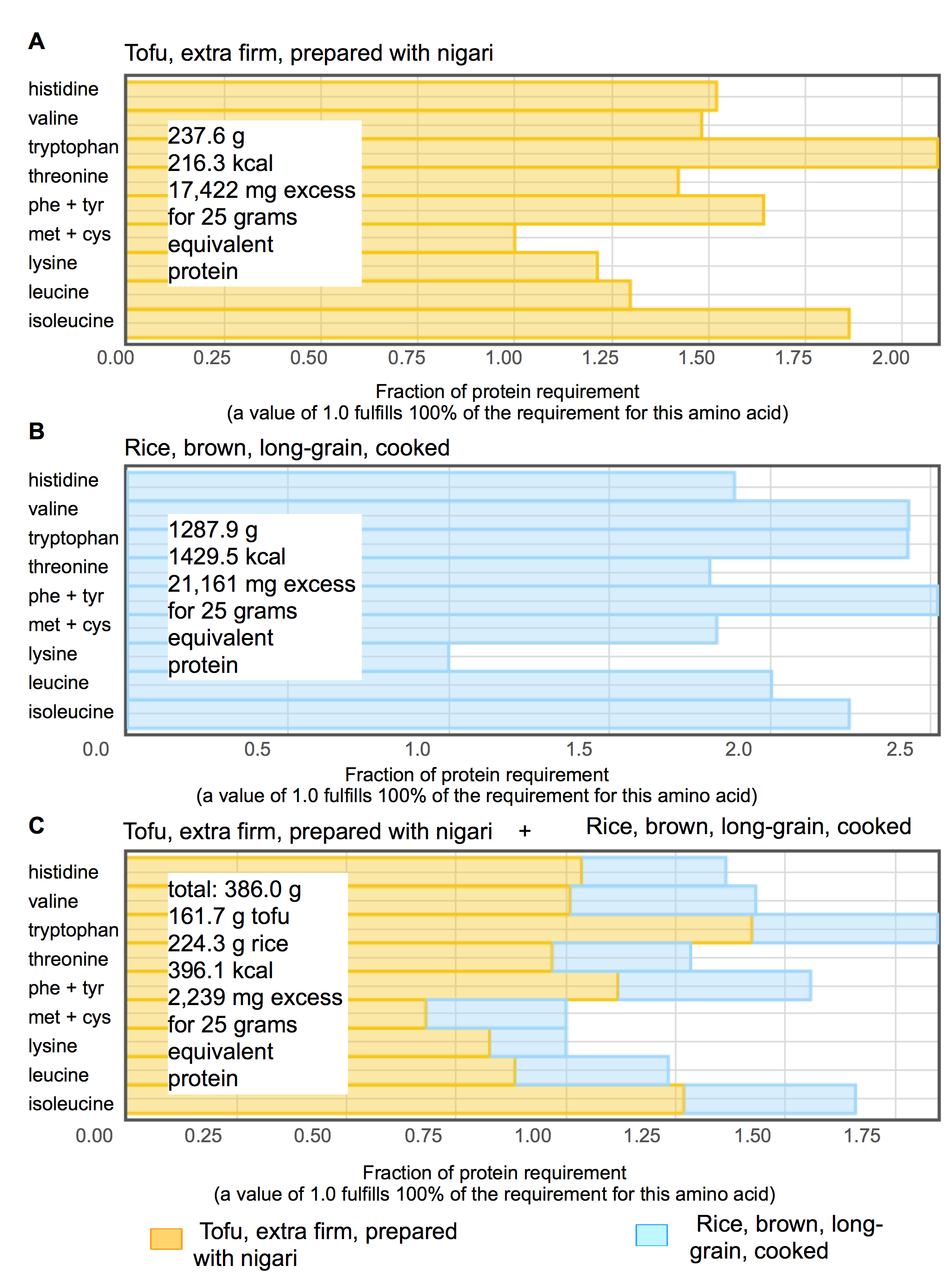

Supplement: Figure S2 — An example pairing of tofu and brown rice. (A) IAA profile of tofu and the corresponding one-way optimized result to obtain 25 grams of high quality protein. (B) IAA profile of brown rice and the corresponding one-way optimized result to obtain 25 grams of high quality protein. (C) IAA profile of the optimized combination of tofu and brown rice to obtain 25 grams of high quality protein. Note that the optimization result in C minimized the excess IAA concentration resulting in a maximally efficient IAA usage. This combination optimized for IAA efficiency is less weight efficient and less calorie efficient than tofu alone (386 g vs 237 g, and 398 kcal vs 216 kcal). (TIFF) [file pone.0018836.s002.tiff]

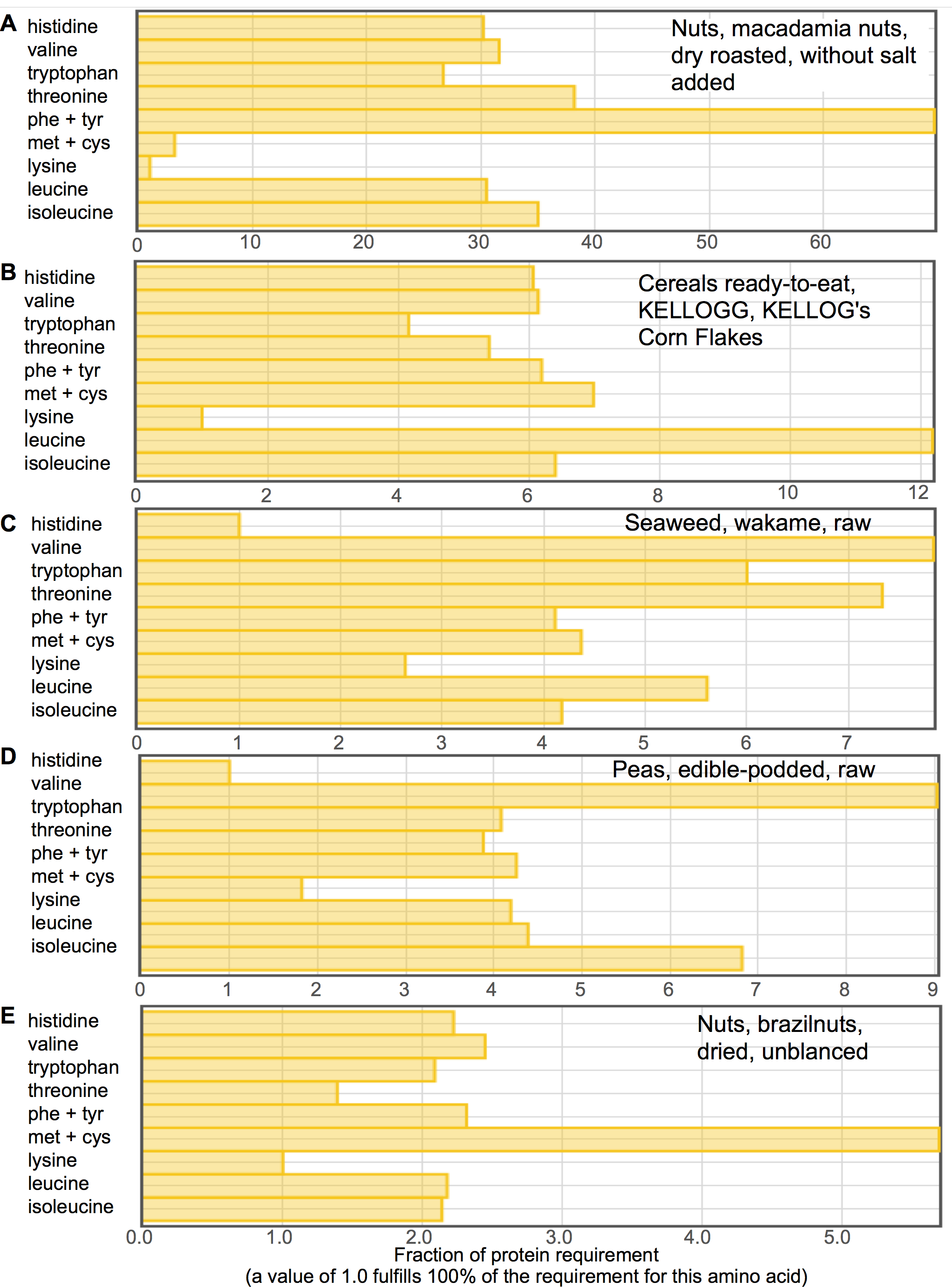

Supplement: Figure S3 — A sampling of amino acid profiles of some of the particularly unbalanced foods listed in Table 2. (TIFF) [file pone.0018836.s003.tiff]
