# Supplementary material for: vProtein: Identifying Optimal Amino Acid Complements from Plant-Based Foods
Source: PLoS One. 2011 Apr 22;6(4):e18836. doi: 10.1371/journal.pone.0018836 (PMC3081312; doi:10.1371/journal.pone.0018836)
Supplement: Table S1 — Minimum Excess: Overall food group based pairings, chi-square statistics, and top-food matches. (DOCX) [file pone.0018836.s004.docx]

**Table S1.** Minimum Excess: Overall food group based pairings, chi-square statistics, and top-food matches.

Spices and Herbs

--overall chi-square: 0.0450135384848 degrees of freedom: 8.0

Vegetables and Vegetable Products 6106 localChiSquare: 0.02034028946 degrees of freedom: 1

Asparagus, frozen, cooked, boiled, drained, with salt 33

Squash, zucchini, baby, raw 30

Asparagus, canned, no salt added, solids and liquids 29

Edamame, frozen, unprepared 28

Lambsquarters, raw 27

Legumes and Legume Products 2092 localChiSquare: 0.00676871023401 degrees of freedom: 1

Soy sauce made from soy (tamari) 25

Winged beans, mature seeds, raw 23

Soy sauce made from soy and wheat (shoyu) 19

Tofu, soft, prepared with calcium sulfate and magnesium chloride (nigari) 19

Tofu, dried-frozen (koyadofu) 19

Tofu, hard, prepared with nigari 19

MORI-NU, Tofu, silken, extra firm 19

Cereal Grains and Pasta 1026 localChiSquare: 0.0114004160279 degrees of freedom: 1

Wheat germ, crude 27

Buckwheat groats, roasted, dry 12

Buckwheat flour, whole-groat 12

Buckwheat groats, roasted, cooked 10

Quinoa, uncooked 10

Wheat bran, crude 10

Quinoa, cooked 10

Oat bran, raw 9

Oat bran, cooked 9

Barley malt flour 9

Fruits and Fruit Juices 917 localChiSquare: 0.00759146381893 degrees of freedom: 1

Applesauce, canned, unsweetened, with added ascorbic acid 15

Peaches, raw 13

Applesauce, canned, sweetened, without salt (includes USDA commodity) 12

Orange juice, chilled, includes from concentrate 12

Orange juice, chilled, includes from concentrate, fortified with calcium and vitamin D 12

Orange juice, chilled, includes from concentrate, fortified with calcium 12

Pineapple, canned, water pack, solids and liquids 12

Nut and Seed Products 904 localChiSquare: 0.00587737031174 degrees of freedom: 1

Nuts, coconut meat, dried (desiccated), sweetened, flaked, packaged 17

Seeds, pumpkin and squash seeds, whole, roasted, without salt 13

Seeds, pumpkin and squash seeds, whole, roasted, with salt added 13

Seeds, breadfruit seeds, roasted 12

Seeds, sisymbrium sp. seeds, whole, dried 12

Seeds, sunflower seed flour, partially defatted 11

Nuts, cashew nuts, oil roasted, without salt added 11

Nuts, cashew nuts, raw 11

Nuts, ginkgo nuts, dried 11

Nuts, cashew nuts, oil roasted, with salt added 11

Breakfast Cereals 453 localChiSquare: 0.00485895102324 degrees of freedom: 1

Cereals ready-to-eat, wheat germ, toasted, plain 28

Cereals ready-to-eat, Ralston Crispy Hexagons 9

Cereals ready-to-eat, USDA Commodity Corn and Rice (includes all commodity brands) 9

Cereals ready-to-eat, GENERAL MILLS, KIX 8

Cereals ready-to-eat, KELLOGG, KELLOGG'S CRISPIX 8

Cereals, oats, regular and quick and instant, unenriched, cooked with water (includes boiling and microwaving), without salt 8

Cereals, corn grits, white, regular and quick, unenriched, dry 8

Cereals, corn grits, yellow, regular and quick, enriched, dry 8

Spices and Herbs 210 localChiSquare: 0.000478736819785 degrees of freedom: 1

Spices, parsley, dried 20

Spices, fenugreek seed 20

Spices, cinnamon, ground 16

Mustard, prepared, yellow 14

Spices, garlic powder 13

Basil, fresh 10

Dill weed, fresh 9

Spices, fennel seed 9

Fats and Oils 44 localChiSquare: 0.000231850249383 degrees of freedom: 1

Salad dressing, french dressing, commercial, regular 9

Salad dressing, italian dressing, reduced fat 9

Sandwich spread, with chopped pickle, regular, unspecified oils 8

Beverages 21 localChiSquare: 0.000623523129811 degrees of freedom: 1

Orange drink, breakfast type, with juice and pulp, frozen concentrate 6

Coffee, instant, decaffeinated, powder 5

Fats and Oils

--overall chi-square: 0.318090889038 degrees of freedom: 8.0

Vegetables and Vegetable Products 1144 localChiSquare: 0.213937823279 degrees of freedom: 1

Peas and carrots, frozen, cooked, boiled, drained, without salt 15

Edamame, frozen, prepared 9

Edamame, frozen, unprepared 8

Lambsquarters, raw 8

Lambsquarters, cooked, boiled, drained, without salt 8

Squash, summer, crookneck and straightneck, raw 8

Squash, summer, crookneck and straightneck, canned, drained, solid, without salt 8

Squash, summer, scallop, raw 8

Fruits and Fruit Juices 218 localChiSquare: 0.00646296547679 degrees of freedom: 1

Pineapple, frozen, chunks, sweetened 6

Avocados, raw, Florida 5

Apricots, canned, juice pack, with skin, solids and liquids 4

Apricots, canned, heavy syrup pack, without skin, solids and liquids 4

Apricots, canned, extra heavy syrup pack, without skin, solids and liquids 4

Apricots, dried, sulfured, stewed, with added sugar 4

Avocados, raw, all commercial varieties 4

Avocados, raw, California 4

Oranges, raw, all commercial varieties 4

Oranges, raw, California, valencias 4

Legumes and Legume Products 170 localChiSquare: 0.0180243340965 degrees of freedom: 1

Falafel, home-prepared 6

Hummus, home prepared 5

Chili with beans, canned 4

Peas, split, mature seeds, raw 4

Peas, split, mature seeds, cooked, boiled, without salt 4

Chickpeas (garbanzo beans, bengal gram), mature seeds, cooked, boiled, with salt 4

Peas, split, mature seeds, cooked, boiled, with salt 4

Nut and Seed Products 64 localChiSquare: 0.0425897032256 degrees of freedom: 1

Seeds, pumpkin and squash seeds, whole, roasted, without salt 6

Nuts, coconut meat, dried (desiccated), sweetened, flaked, packaged 3

Nuts, formulated, wheat-based, flavored, macadamia flavored, without salt 3

Seeds, lotus seeds, raw 3

Nuts, chestnuts, european, raw, unpeeled 2

Nuts, chestnuts, european, raw, peeled 2

Nuts, chestnuts, european, dried, unpeeled 2

Nuts, chestnuts, european, dried, peeled 2

Nuts, chestnuts, european, boiled and steamed 2

Nuts, formulated, wheat-based, unflavored, with salt added 2

Spices and Herbs 44 localChiSquare: 0.0079598514107 degrees of freedom: 1

Spices, garlic powder 9

Spices, cinnamon, ground 8

Spices, mustard seed, ground 7

Cereal Grains and Pasta 27 localChiSquare: 0.105037512659 degrees of freedom: 1

Wheat germ, crude 10

Breakfast Cereals 17 localChiSquare: 0.038062134053 degrees of freedom: 1

Cereals ready-to-eat, wheat germ, toasted, plain 10

Cereals, CREAM OF WHEAT, 1 minute cook time, cooked with water, stove-top, without salt 2

Cereals, oats, instant, fortified, plain, dry 1

Fats and Oils 4 localChiSquare: 0.00122771760406 degrees of freedom: 1

Salad dressing, french dressing, reduced fat 2

Breakfast Cereals

--overall chi-square: 0.320955907802 degrees of freedom: 8.0

Vegetables and Vegetable Products 10324 localChiSquare: 0.093118355101 degrees of freedom: 1

Asparagus, frozen, cooked, boiled, drained, with salt 168

Asparagus, canned, no salt added, solids and liquids 164

Cauliflower, green, raw 141

Legumes and Legume Products 4298 localChiSquare: 0.0808919977811 degrees of freedom: 1

Soy sauce made from soy (tamari) 85

Tofu, dried-frozen (koyadofu) 74

Tofu, raw, regular, prepared with calcium sulfate 74

Tofu, dried-frozen (koyadofu), prepared with calcium sulfate 74

Tofu, soft, prepared with calcium sulfate and magnesium chloride (nigari) 73

Winged beans, mature seeds, raw 73

Tofu, extra firm, prepared with nigari 73

Soybeans, mature seeds, cooked, boiled, with salt 73

Fruits and Fruit Juices 1114 localChiSquare: 0.0169749280918 degrees of freedom: 1

Pineapple, frozen, chunks, sweetened 82

Applesauce, canned, unsweetened, with added ascorbic acid 67

Watermelon, raw 52

Applesauce, canned, sweetened, with salt 52

Applesauce, canned, sweetened, without salt (includes USDA commodity) 51

Spices and Herbs 453 localChiSquare: 0.00830998955141 degrees of freedom: 1

Spices, fenugreek seed 140

Spices, garlic powder 95

Spices, parsley, dried 72

Spices, cinnamon, ground 66

Spices, onion powder 46

Dill weed, fresh 7

Spices, mustard seed, ground 4

Spearmint, fresh 3

Mustard, prepared, yellow 3

Rosemary, fresh 2

Cereal Grains and Pasta 436 localChiSquare: 0.0873943550608 degrees of freedom: 1

Wheat germ, crude 133

Oat bran, raw 4

Oat bran, cooked 3

Wheat bran, crude 3

Amaranth, uncooked 2

Nut and Seed Products 304 localChiSquare: 0.0750691918921 degrees of freedom: 1

Nuts, coconut meat, dried (desiccated), sweetened, flaked, packaged 26

Seeds, lotus seeds, raw 24

Seeds, lotus seeds, dried 18

Seeds, pumpkin and squash seeds, whole, roasted, with salt added 11

Seeds, pumpkin and squash seeds, whole, roasted, without salt 10

Seeds, breadfruit seeds, raw 6

Breakfast Cereals 268 localChiSquare: 0.0291932341819 degrees of freedom: 1

Cereals ready-to-eat, wheat germ, toasted, plain 134

Cereals, CREAM OF WHEAT, instant, prepared with water, with salt, (wheat) 2

Fats and Oils 17 localChiSquare: 0.00303229282333 degrees of freedom: 1

Salad dressing, french dressing, reduced fat 4

Margarine, regular, hard, soybean (hydrogenated) 3

Margarine-like, vegetable oil spread, unspecified oils, approximately 37 fat, with salt 3

Salad dressing, italian dressing, reduced fat 3

Sandwich spread, with chopped pickle, regular, unspecified oils 2

Beverages 8 localChiSquare: 0.00233835229034 degrees of freedom: 1

Coffee, instant, decaffeinated, powder 2

Fruits and Fruit Juices

--overall chi-square: 0.116805960121 degrees of freedom: 8.0

Vegetables and Vegetable Products 20733 localChiSquare: 0.0181941117293 degrees of freedom: 1

Cauliflower, green, cooked, with salt 165

Cauliflower, green, cooked, no salt added 164

Cauliflower, green, raw 162

Squash, zucchini, baby, raw 158

Cauliflower, cooked, boiled, drained, with salt 155

Cauliflower, frozen, cooked, boiled, drained, with salt 155

Legumes and Legume Products 8921 localChiSquare: 0.0422309570388 degrees of freedom: 1

Falafel, home-prepared 119

Soy flour, defatted 107

Soy flour, low-fat, crude protein basis (N x 6.25) 107

Soy flour, defatted, crude protein basis (N x 6.25) 106

Soy flour, full-fat, roasted 104

Chickpeas (garbanzo beans, bengal gram), mature seeds, cooked, boiled, with salt 104

Soy flour, full-fat, roasted, crude protein basis (N x 6.25) 104

Soybeans, mature seeds, dry roasted 103

Soy meal, defatted, raw 103

Nut and Seed Products 3528 localChiSquare: 0.00164662793847 degrees of freedom: 1

Seeds, pumpkin and squash seeds, whole, roasted, without salt 138

Seeds, pumpkin and squash seeds, whole, roasted, with salt added 138

Nuts, coconut meat, dried (desiccated), sweetened, flaked, packaged 107

Nuts, formulated, wheat-based, flavored, macadamia flavored, without salt 98

Nuts, formulated, wheat-based, all flavors except macadamia, without salt 88

Nuts, formulated, wheat-based, unflavored, with salt added 83

Seeds, lotus seeds, raw 82

Cereal Grains and Pasta 2668 localChiSquare: 0.0291092648903 degrees of freedom: 1

Wheat germ, crude 144

Quinoa, uncooked 94

Quinoa, cooked 93

Amaranth, uncooked 70

Fruits and Fruit Juices 2102 localChiSquare: 0.0292806607677 degrees of freedom: 1

Applesauce, canned, unsweetened, with added ascorbic acid 61

Dates, medjool 55

Pineapple, frozen, chunks, sweetened 51

Pineapple, canned, juice pack, solids and liquids 50

Dates, deglet noor 46

Pineapple, canned, light syrup pack, solids and liquids 46

Breakfast Cereals 1114 localChiSquare: 0.0137955621516 degrees of freedom: 1

Cereals ready-to-eat, wheat, shredded, plain, sugar and salt free 40

Cereals ready-to-eat, KELLOGG, KELLOGG'S ALL-BRAN Original 27

Incaparina, dry mix (corn and soy flours), unprepared 26

Cereals, oats, instant, fortified, plain, dry 24

Cereals, farina, enriched, assorted brands including CREAM OF WHEAT, quick (1-3 minutes), dry 21

Cereals, oats, instant, fortified, plain, prepared with water (boiling water added or microwaved) 20

Spices and Herbs 917 localChiSquare: 0.00387329283948 degrees of freedom: 1

Spices, fenugreek seed 154

Spices, cinnamon, ground 97

Spices, garlic powder 75

Spices, caraway seed 67

Mustard, prepared, yellow 56

Spices, poppy seed 49

Basil, fresh 28

Fats and Oils 218 localChiSquare: 8.1814366329e-05 degrees of freedom: 1

Salad dressing, italian dressing, reduced fat 68

Margarine-like, vegetable oil spread, unspecified oils, approximately 37 fat, with salt 61

Margarine, regular, hard, soybean (hydrogenated) 56

Salad dressing, french dressing, reduced fat 23

Sandwich spread, with chopped pickle, regular, unspecified oils 7

Beverages 73 localChiSquare: 0.000598250961578 degrees of freedom: 1

Coffee, instant, decaffeinated, powder 23

Vegetables and Vegetable Products

--overall chi-square: 0.0451640379861 degrees of freedom: 8.0

Vegetables and Vegetable Products 143356 localChiSquare: 0.0311608211534 degrees of freedom: 1

Squash, zucchini, baby, raw 1129

Squash, summer, zucchini, includes skin, frozen, cooked, boiled, drained, with salt 958

Squash, summer, zucchini, includes skin, raw 915

Squash, summer, zucchini, includes skin, frozen, unprepared 908

Squash, summer, all varieties, raw 893

Squash, summer, scallop, cooked, boiled, drained, without salt 876

Squash, summer, scallop, raw 875

Squash, summer, scallop, cooked, boiled, drained, with salt 873

Lambs quarters, cooked, boiled, drained, with salt 869

Legumes and Legume Products 39440 localChiSquare: 9.85770900549e-06 degrees of freedom: 1

MORI-NU, Tofu, silken, soft 480

Soy sauce made from soy and wheat (shoyu) 476

Soy sauce made from soy and wheat (shoyu), low sodium 476

Soy flour, low-fat, crude protein basis (N x 6.25) 390

Soy flour, defatted, crude protein basis (N x 6.25) 387

Soybeans, mature seeds, cooked, boiled, with salt 383

Soy meal, defatted, raw, crude protein basis (N x 6.25) 382

Cereal Grains and Pasta 23558 localChiSquare: 0.0108515228085 degrees of freedom: 1

Wheat germ, crude 757

Oat bran, cooked 343

Oat bran, raw 326

Wheat bran, crude 303

Barley malt flour 301

Quinoa, cooked 297

Quinoa, uncooked 294

Amaranth, uncooked 292

Nut and Seed Products 22523 localChiSquare: 0.00271891024592 degrees of freedom: 1

Nuts, coconut meat, dried (desiccated), sweetened, flaked, packaged 639

Seeds, pumpkin and squash seeds, whole, roasted, with salt added 498

Seeds, pumpkin and squash seeds, whole, roasted, without salt 497

Seeds, sunflower seed flour, partially defatted 373

Seeds, cottonseed kernels, roasted (glandless) 354

Seeds, sesame flour, low-fat 329

Nuts, acorns, dried 322

Fruits and Fruit Juices 20733 localChiSquare: 0.0078454646961 degrees of freedom: 1

Pineapple, frozen, chunks, sweetened 368

Pineapple, canned, juice pack, solids and liquids 331

Peaches, raw 314

Pineapple, canned, water pack, solids and liquids 292

Cherries, sweet, raw 285

Pineapple, canned, extra heavy syrup pack, solids and liquids 257

Pineapple, canned, heavy syrup pack, solids and liquids 253

Pineapple, canned, light syrup pack, solids and liquids 252

Breakfast Cereals 10324 localChiSquare: 0.0048054060739 degrees of freedom: 1

Cereals ready-to-eat, wheat germ, toasted, plain 714

Cereals, oats, regular and quick and instant, unenriched, cooked with water (includes boiling and microwaving), without salt 196

Cereals, oats, instant, fortified, plain, prepared with water (boiling water added or microwaved) 196

Incaparina, dry mix (corn and soy flours), unprepared 183

Cereals, oats, instant, fortified, plain, dry 179

Cereals, farina, enriched, cooked with water, without salt 178

Cereals, oats, regular and quick and instant, not fortified, dry 174

Spices and Herbs 6106 localChiSquare: 0.00391396520643 degrees of freedom: 1

Mustard, prepared, yellow 664

Basil, fresh 421

Spices, garlic powder 418

Spices, fenugreek seed 395

Spices, cinnamon, ground 387

Spices, caraway seed 369

Spices, parsley, dried 352

Spices, onion powder 285

Fats and Oils 1144 localChiSquare: 5.56791916488e-05 degrees of freedom: 1

Sandwich spread, with chopped pickle, regular, unspecified oils 235

Beverages 519 localChiSquare: 0.000493870882981 degrees of freedom: 1

Orange drink, breakfast type, with juice and pulp, frozen concentrate 155

Coffee, instant, regular, powder 122

Coffee, instant, decaffeinated, powder 121

Nut and Seed Products

--overall chi-square: 0.38751080118 degrees of freedom: 8.0

Vegetables and Vegetable Products 22523 localChiSquare: 0.0525468620215 degrees of freedom: 1

Lambsquarters, raw 293

Lambsquarters, cooked, boiled, drained, without salt 284

Lambs quarters, cooked, boiled, drained, with salt 284

Peas and carrots, frozen, cooked, boiled, drained, without salt 220

Asparagus, frozen, cooked, boiled, drained, with salt 213

Cauliflower, green, cooked, no salt added 207

Cauliflower, green, cooked, with salt 207

Edamame, frozen, prepared 204

Legumes and Legume Products 11660 localChiSquare: 0.160304737429 degrees of freedom: 1

Soy sauce made from soy (tamari) 180

Peas, split, mature seeds, cooked, boiled, with salt 151

Tofu, raw, firm, prepared with calcium sulfate 144

Falafel, home-prepared 143

MOR-NU, Tofu, silken, lite firm 141

MORI-NU, Tofu, silken, lite extra firm 140

Tofu, soft, prepared with calcium sulfate and magnesium chloride (nigari) 137

Tofu, extra firm, prepared with nigari 137

Tofu, hard, prepared with nigari 137

Fruits and Fruit Juices 3528 localChiSquare: 0.00293013168432 degrees of freedom: 1

Apples, frozen, unsweetened, heated 140

Applesauce, canned, unsweetened, without added ascorbic acid (includes USDA commodity) 137

Applesauce, canned, unsweetened, with added ascorbic acid 137

Applesauce, canned, sweetened, without salt (includes USDA commodity) 115

Applesauce, canned, sweetened, with salt 115

Apples, dried, sulfured, stewed, with added sugar 96

Orange juice, chilled, includes from concentrate 96

Orange juice, chilled, includes from concentrate, fortified with calcium and vitamin D 96

Spices and Herbs 904 localChiSquare: 0.00364878727625 degrees of freedom: 1

Spices, fenugreek seed 230

Spices, garlic powder 134

Spices, cinnamon, ground 96

Dill weed, fresh 81

Mustard, prepared, yellow 37

Spices, ginger, ground 13

Nut and Seed Products 714 localChiSquare: 0.0747860775898 degrees of freedom: 1

Seeds, pumpkin and squash seeds, whole, roasted, without salt 70

Seeds, pumpkin and squash seeds, whole, roasted, with salt added 70

Seeds, lotus seeds, dried 53

Seeds, lotus seeds, raw 50

Nuts, coconut meat, dried (desiccated), sweetened, flaked, packaged 46

Seeds, breadfruit seeds, raw 24

Cereal Grains and Pasta 394 localChiSquare: 0.117605817335 degrees of freedom: 1

Wheat germ, crude 193

Amaranth, uncooked 7

Breakfast Cereals 304 localChiSquare: 0.0424705599446 degrees of freedom: 1

Cereals ready-to-eat, wheat germ, toasted, plain 196

Incaparina, dry mix (corn and soy flours), unprepared 17

Cereals ready-to-eat, MALT-O-MEAL, GOLDEN PUFFS 7

Cereals ready-to-eat, GENERAL MILLS, CINNAMON TOAST CRUNCH 5

Cereals, oats, instant, fortified, plain, prepared with water (boiling water added or microwaved) 5

Cereals ready-to-eat, KELLOGG, KELLOGG'S FROSTED FLAKES 4

Cereals, oats, instant, fortified, plain, dry 4

Fats and Oils 64 localChiSquare: 0.00213851898354 degrees of freedom: 1

Margarine, regular, hard, soybean (hydrogenated) 20

Salad dressing, french dressing, reduced fat 17

Margarine-like, vegetable oil spread, unspecified oils, approximately 37 fat, with salt 15

Sandwich spread, with chopped pickle, regular, unspecified oils 4

Beverages 6 localChiSquare: 0.00290936587385 degrees of freedom: 1

Orange drink, breakfast type, with juice and pulp, frozen concentrate 4

Coffee, instant, with chicory, powder 2

Beverages

--overall chi-square: 0.418541321691 degrees of freedom: 8.0

Vegetables and Vegetable Products 519 localChiSquare: 0.0727403905458 degrees of freedom: 1

Asparagus, frozen, cooked, boiled, drained, without salt 9

Cauliflower, cooked, boiled, drained, with salt 8

Cauliflower, cooked, boiled, drained, without salt 8

Cauliflower, frozen, unprepared 8

Cauliflower, green, raw 8

Cauliflower, green, cooked, no salt added 8

Legumes and Legume Products 257 localChiSquare: 0.154329450937 degrees of freedom: 1

Soy sauce made from soy (tamari) 6

Falafel, home-prepared 6

Peas, split, mature seeds, raw 5

Chickpeas (garbanzo beans, bengal gram), mature seeds, cooked, boiled, with salt 5

Peas, split, mature seeds, cooked, boiled, without salt 5

Peas, split, mature seeds, cooked, boiled, with salt 5

Tofu, raw, regular, prepared with calcium sulfate 5

Fruits and Fruit Juices 73 localChiSquare: 0.00550924439145 degrees of freedom: 1

Apples, frozen, unsweetened, heated 7

Apples, dried, sulfured, uncooked 6

Watermelon, raw 6

Apples, dehydrated (low moisture), sulfured, uncooked 5

Apples, canned, sweetened, sliced, drained, unheated 3

Apples, canned, sweetened, sliced, drained, heated 3

Apples, dehydrated (low moisture), sulfured, stewed 3

Apples, dried, sulfured, stewed, without added sugar 3

Apples, dried, sulfured, stewed, with added sugar 3

Apples, frozen, unsweetened, unheated 3

Spices and Herbs 21 localChiSquare: 0.00470129056794 degrees of freedom: 1

Spices, fenugreek seed 9

Spices, cinnamon, ground 4

Spices, parsley, dried 4

Spices, garlic powder 3

Breakfast Cereals 8 localChiSquare: 0.0399846403047 degrees of freedom: 1

Cereals ready-to-eat, wheat germ, toasted, plain 8

Cereal Grains and Pasta 8 localChiSquare: 0.119409142771 degrees of freedom: 1

Wheat germ, crude 8

Nut and Seed Products 6 localChiSquare: 0.0963756924588 degrees of freedom: 1

Seeds, lotus seeds, raw 2

Seeds, lotus seeds, dried 2

Seeds, pumpkin and squash seeds, whole, roasted, without salt 1

Legumes and Legume Products

--overall chi-square: 0.102719975733 degrees of freedom: 8.0

Vegetables and Vegetable Products 39440 localChiSquare: 0.00492702283856 degrees of freedom: 1

Carrots, canned, no salt added, solids and liquids 348

Carrots, cooked, boiled, drained, with salt 338

Tomatoes, sun-dried 331

Tomatoes, yellow, raw 325

Tomatoes, orange, raw 321

Nut and Seed Products 11660 localChiSquare: 0.0205150845021 degrees of freedom: 1

Nuts, chestnuts, japanese, dried 318

Nuts, chestnuts, japanese, roasted 307

Nuts, chestnuts, japanese, raw 303

Nuts, chestnuts, japanese, boiled and steamed 293

Nuts, chestnuts, european, dried, peeled 288

Nuts, chestnuts, european, roasted 281

Nuts, chestnuts, european, dried, unpeeled 278

Nuts, chestnuts, european, boiled and steamed 258

Cereal Grains and Pasta 11135 localChiSquare: 0.00186850937232 degrees of freedom: 1

Quinoa, cooked 207

Barley malt flour 193

Macaroni, protein-fortified, dry, enriched, (n x 6.25) 165

Spaghetti, protein-fortified, dry, enriched (n x 6.25) 164

Fruits and Fruit Juices 8921 localChiSquare: 0.000227765942843 degrees of freedom: 1

Peaches, raw 291

Pineapple, canned, water pack, solids and liquids 272

Pineapple, canned, juice pack, solids and liquids 264

Kiwifruit, gold, raw 252

Pineapple, raw, all varieties 238

Pineapple, canned, light syrup pack, solids and liquids 233

Pineapple, canned, extra heavy syrup pack, solids and liquids 233

Pineapple, canned, heavy syrup pack, solids and liquids 229

Breakfast Cereals 4298 localChiSquare: 5.46568714738e-05 degrees of freedom: 1

Cereals, oats, instant, fortified, plain, dry 176

Cereals, oats, regular and quick and instant, unenriched, cooked with water (includes boiling and microwaving), without salt 145

Cereals, oats, regular and quick and instant, not fortified, dry 105

Cereals, oats, instant, fortified, plain, prepared with water (boiling water added or microwaved) 99

Cereals ready-to-eat, wheat, shredded, plain, sugar and salt free 67

Cereals, farina, unenriched, dry 60

Cereals, CREAM OF WHEAT, regular (10 minute), cooked with water, without salt 58

Cereals, CREAM OF WHEAT, instant, dry 58

Legumes and Legume Products 3746 localChiSquare: 0.0832819741993 degrees of freedom: 1

MORI-NU, Tofu, silken, soft 206

Soy sauce made from soy and wheat (shoyu) 195

Soy sauce made from soy and wheat (shoyu), low sodium 195

Peanut flour, defatted 194

Peanut flour, low fat 110

Hummus, home prepared 90

Peanuts, virginia, raw 45

Peanuts, valencia, raw 44

Peanuts, valencia, oil-roasted, with salt 44

Peanuts, virginia, oil-roasted, with salt 44

Spices and Herbs 2092 localChiSquare: 0.007293360563 degrees of freedom: 1

Spices, cinnamon, ground 304

Mustard, prepared, yellow 251

Spices, garlic powder 210

Spices, caraway seed 199

Basil, fresh 174

Spices, poppy seed 81

Spices, fennel seed 58

Beverages 257 localChiSquare: 7.06496397655e-07 degrees of freedom: 1

Orange drink, breakfast type, with juice and pulp, frozen concentrate 90

Coffee, instant, decaffeinated, powder 57

Fats and Oils 170 localChiSquare: 0.00153903493252 degrees of freedom: 1

Salad dressing, italian dressing, reduced fat 85

Sandwich spread, with chopped pickle, regular, unspecified oils 49

Cereal Grains and Pasta

--overall chi-square: 0.399417416318 degrees of freedom: 8.0

Vegetables and Vegetable Products 23558 localChiSquare: 0.0835215385262 degrees of freedom: 1

Lambsquarters, raw 356

Lambsquarters, cooked, boiled, drained, without salt 338

Lambs quarters, cooked, boiled, drained, with salt 338

Cauliflower, green, raw 334

Cauliflower, green, cooked, no salt added 330

Cauliflower, green, cooked, with salt 330

Asparagus, canned, no salt added, solids and liquids 323

Cauliflower, frozen, unprepared 322

Cauliflower, cooked, boiled, drained, with salt 322

Legumes and Legume Products 11135 localChiSquare: 0.135977091449 degrees of freedom: 1

Soy protein isolate, PROTEIN TECHNOLOGIES INTERNATIONAL, SUPRO 192

Soy protein isolate, potassium type, crude protein basis 191

Soy protein concentrate, crude protein basis (N x 6.25), produced by acid wash 190

Soy protein isolate, potassium type 190

Soy protein concentrate, produced by acid wash 189

MORI-NU, Tofu, silken, extra firm 187

MORI-NU, Tofu, silken, lite extra firm 187

Soy protein isolate, PROTEIN TECHNOLOGIES INTERNATIONAL, ProPlus 187

Fruits and Fruit Juices 2668 localChiSquare: 0.0150753917316 degrees of freedom: 1

Pineapple, frozen, chunks, sweetened 174

Applesauce, canned, unsweetened, without added ascorbic acid (includes USDA commodity) 173

Applesauce, canned, unsweetened, with added ascorbic acid 173

Applesauce, canned, sweetened, without salt (includes USDA commodity) 146

Applesauce, canned, sweetened, with salt 146

Watermelon, raw 131

Spices and Herbs 1026 localChiSquare: 0.00751623979826 degrees of freedom: 1

Spices, fenugreek seed 339

Spices, parsley, dried 179

Spices, garlic powder 179

Spices, cinnamon, ground 149

Spices, onion powder 109

Dill weed, fresh 26

Mustard, prepared, yellow 16

Spices, mustard seed, ground 11

Spices, ginger, ground 3

Rosemary, fresh 2

Cereal Grains and Pasta 580 localChiSquare: 0.107895871132 degrees of freedom: 1

Wheat germ, crude 288

Rice, white, medium-grain, raw, enriched 3

Quinoa, cooked 3

Rice, white, medium-grain, raw, unenriched 3

Amaranth, uncooked 2

Breakfast Cereals 436 localChiSquare: 0.0365702182878 degrees of freedom: 1

Cereals ready-to-eat, wheat germ, toasted, plain 298

Incaparina, dry mix (corn and soy flours), unprepared 7

Cereals, CREAM OF WHEAT, instant, prepared with water, with salt, (wheat) 2

Nut and Seed Products 394 localChiSquare: 0.0899296988244 degrees of freedom: 1

Nuts, coconut meat, dried (desiccated), sweetened, flaked, packaged 87

Seeds, lotus seeds, raw 37

Seeds, lotus seeds, dried 22

Seeds, breadfruit seeds, boiled 13

Seeds, pumpkin and squash seeds, whole, roasted, without salt 13

Seeds, breadfruit seeds, raw 12

Fats and Oils 27 localChiSquare: 0.00354574650995 degrees of freedom: 1

Salad dressing, french dressing, reduced fat 10

Margarine, regular, hard, soybean (hydrogenated) 6

Margarine-like, vegetable oil spread, unspecified oils, approximately 37 fat, with salt 5

Sandwich spread, with chopped pickle, regular, unspecified oils 2

Beverages 8 localChiSquare: 0.0028122649222 degrees of freedom: 1

Coffee, instant, decaffeinated, powder 2
