# Supplementary material for: vProtein: Identifying Optimal Amino Acid Complements from Plant-Based Foods
Source: PLoS One. 2011 Apr 22;6(4):e18836. doi: 10.1371/journal.pone.0018836 (PMC3081312; doi:10.1371/journal.pone.0018836)
Supplement: Table S2 — Minimum Calories: Overall food group based pairings, chi-square statistics, and top-food matches. (DOCX) [file pone.0018836.s005.docx]

**Table S2.** Minimum Calories: Overall food group based pairings, chi-square statistics, and top-food matches.

Spices and Herbs

--overall chi-square: 0.127983594959 degrees of freedom: 8.0

Vegetables and Vegetable Products 3154 localChiSquare: 0.0437105756575 degrees of freedom: 1

Edamame, frozen, unprepared 20

Lambsquarters, raw 20

Squash, zucchini, baby, raw 20

Lambsquarters, cooked, boiled, drained, without salt 19

Lambs quarters, cooked, boiled, drained, with salt 19

Edamame, frozen, prepared 18

Asparagus, frozen, cooked, boiled, drained, with salt 18

Spinach, frozen, chopped or leaf, cooked, boiled, drained, without salt 16

Legumes and Legume Products 1222 localChiSquare: 0.0337375944858 degrees of freedom: 1

Winged beans, mature seeds, raw 16

Soy sauce made from soy (tamari) 15

MORI-NU, Tofu, silken, soft 15

Tofu, soft, prepared with calcium sulfate and magnesium chloride (nigari) 14

Tofu, dried-frozen (koyadofu) 14

Tofu, hard, prepared with nigari 14

MORI-NU, Tofu, silken, extra firm 14

MOR-NU, Tofu, silken, lite firm 14

MORI-NU, Tofu, silken, lite extra firm 14

Cereal Grains and Pasta 375 localChiSquare: 0.0297931411587 degrees of freedom: 1

Wheat germ, crude 12

Oat bran, raw 5

Oat bran, cooked 5

Wheat bran, crude 5

Amaranth, uncooked 4

Nut and Seed Products 358 localChiSquare: 0.0153594015368 degrees of freedom: 1

Seeds, cottonseed flour, partially defatted (glandless) 9

Seeds, cottonseed flour, low fat (glandless) 9

Seeds, cottonseed meal, partially defatted (glandless) 9

Seeds, breadfruit seeds, boiled 8

Seeds, sunflower seed flour, partially defatted 8

Seeds, breadfruit seeds, roasted 8

Seeds, pumpkin and squash seeds, whole, roasted, without salt 8

Seeds, pumpkin and squash seeds, whole, roasted, with salt added 8

Nuts, ginkgo nuts, dried 7

Seeds, cottonseed kernels, roasted (glandless) 7

Fruits and Fruit Juices 343 localChiSquare: 0.0212023362316 degrees of freedom: 1

Cranberries, raw 7

Watermelon, raw 7

Rhubarb, raw 6

Lime juice, raw 5

Orange juice, chilled, includes from concentrate 5

Orange juice, chilled, includes from concentrate, fortified with calcium and vitamin D 5

Orange juice, chilled, includes from concentrate, fortified with calcium 5

Apricots, dehydrated (low-moisture), sulfured, stewed 4

Apricots, frozen, sweetened 4

Cherimoya, raw 4

Breakfast Cereals 155 localChiSquare: 0.0143700619506 degrees of freedom: 1

Cereals ready-to-eat, wheat germ, toasted, plain 13

Incaparina, dry mix (corn and soy flours), unprepared 5

Cereals, oats, regular and quick and instant, unenriched, cooked with water (includes boiling and microwaving), without salt 4

Cereals, CREAM OF WHEAT, 1 minute cook time, cooked with water, microwaved, without salt 4

Cereals, oats, regular and quick and instant, not fortified, dry 3

Cereals, oats, instant, fortified, plain, prepared with water (boiling water added or microwaved) 3

Cereals, CREAM OF WHEAT, instant, prepared with water, with salt, (wheat) 2

Spices and Herbs 90 localChiSquare: 2.24420706712e-05 degrees of freedom: 1

Spices, parsley, dried 14

Spices, fenugreek seed 12

Spices, mustard seed, ground 9

Mustard, prepared, yellow 7

Spices, caraway seed 6

Basil, fresh 6

Spices, oregano, dried 5

Dill weed, fresh 5

Rosemary, fresh 3

Fats and Oils 11 localChiSquare: 0.00172170323173 degrees of freedom: 1

Sandwich spread, with chopped pickle, regular, unspecified oils 2

Beverages 9 localChiSquare: 0.000822778619153 degrees of freedom: 1

Orange drink, breakfast type, with juice and pulp, frozen concentrate 3

Coffee, instant, decaffeinated, powder 2

Fats and Oils

--overall chi-square: 0.437442516486 degrees of freedom: 8.0

Vegetables and Vegetable Products 443 localChiSquare: 0.341545008061 degrees of freedom: 1

Peas and carrots, frozen, cooked, boiled, drained, without salt 6

Squash, summer, crookneck and straightneck, raw 4

Squash, summer, crookneck and straightneck, canned, drained, solid, without salt 4

Squash, summer, crookneck and straightneck, frozen, cooked, boiled, drained, without salt 4

Squash, summer, scallop, raw 4

Squash, summer, zucchini, includes skin, raw 4

Squash, summer, zucchini, includes skin, frozen, cooked, boiled, drained, without salt 4

Squash, summer, all varieties, raw 4

Squash, summer, zucchini, includes skin, frozen, cooked, boiled, drained, with salt 4

Squash, zucchini, baby, raw 4

Legumes and Legume Products 97 localChiSquare: 0.00138318504018 degrees of freedom: 1

Bacon, meatless 3

Meat extender 3

Sausage, meatless 3

Soybeans, mature seeds, raw 3

Soybeans, mature cooked, boiled, without salt 3

Soybeans, mature seeds, roasted, salted 3

Soybeans, mature seeds, dry roasted 3

Soy flour, full-fat, raw 3

Soy flour, full-fat, roasted 3

Soy flour, defatted 3

Nut and Seed Products 22 localChiSquare: 0.0443259329393 degrees of freedom: 1

Seeds, pumpkin and squash seeds, whole, roasted, without salt 6

Fruits and Fruit Juices 14 localChiSquare: 0.0704560831334 degrees of freedom: 1

Avocados, raw, all commercial varieties 3

Spices and Herbs 11 localChiSquare: 0.00067278581042 degrees of freedom: 1

Spices, cinnamon, ground 4

Spices, mustard seed, ground 2

Cereal Grains and Pasta 7 localChiSquare: 0.113781851868 degrees of freedom: 1

Wheat germ, crude 5

Breakfast Cereals 6 localChiSquare: 0.0381846723753 degrees of freedom: 1

Cereals ready-to-eat, wheat germ, toasted, plain 5

Breakfast Cereals

--overall chi-square: 0.744160554109 degrees of freedom: 8.0

Vegetables and Vegetable Products 3928 localChiSquare: 0.00650430697311 degrees of freedom: 1

Horseradish-tree, leafy tips, cooked, boiled, drained, with salt 76

Cauliflower, green, raw 73

Cauliflower, green, cooked, no salt added 73

Cauliflower, green, cooked, with salt 73

Cauliflower, cooked, boiled, drained, with salt 71

Jute, potherb, cooked, boiled, drained, without salt 70

Jute, potherb, cooked, boiled, drained, with salt 70

Legumes and Legume Products 3358 localChiSquare: 0.570718926202 degrees of freedom: 1

Tofu, dried-frozen (koyadofu) 72

Tofu, raw, regular, prepared with calcium sulfate 72

Tofu, dried-frozen (koyadofu), prepared with calcium sulfate 72

Tofu, soft, prepared with calcium sulfate and magnesium chloride (nigari) 71

Winged beans, mature seeds, raw 71

Tofu, extra firm, prepared with nigari 71

Soybeans, mature seeds, cooked, boiled, with salt 71

Soy flour, full-fat, raw, crude protein basis (N x 6.25) 71

Cereal Grains and Pasta 221 localChiSquare: 0.0835899764968 degrees of freedom: 1

Wheat germ, crude 66

Oat bran, raw 3

Oat bran, cooked 2

Wheat bran, crude 2

Amaranth, uncooked 1

Nut and Seed Products 162 localChiSquare: 0.0706252911305 degrees of freedom: 1

Seeds, lotus seeds, raw 23

Seeds, lotus seeds, dried 17

Seeds, pumpkin and squash seeds, whole, roasted, with salt added 9

Seeds, pumpkin and squash seeds, whole, roasted, without salt 8

Seeds, breadfruit seeds, raw 2

Spices and Herbs 155 localChiSquare: 0.00111811680144 degrees of freedom: 1

Spices, fenugreek seed 71

Spices, parsley, dried 70

Spices, mustard seed, ground 2

Spearmint, fresh 2

Rosemary, fresh 1

Breakfast Cereals 134 localChiSquare: 0.0275864239562 degrees of freedom: 1

Cereals ready-to-eat, wheat germ, toasted, plain 67

Cereals, CREAM OF WHEAT, instant, prepared with water, with salt, (wheat) 1

Fruits and Fruit Juices 84 localChiSquare: 0.0946178637655 degrees of freedom: 1

Lime juice, raw 6

Apples, dried, sulfured, stewed, with added sugar 4

Apples, raw, without skin, cooked, microwave 3

Apples, dehydrated (low moisture), sulfured, stewed 3

Apples, frozen, unsweetened, heated 3

Apples, frozen, unsweetened, unheated 2

Tangerines, (mandarin oranges), canned, light syrup pack 2

Tangerine juice, frozen concentrate, sweetened, diluted with 3 volume water 2

Peaches, spiced, canned, heavy syrup pack, solids and liquids 1

Fats and Oils 6 localChiSquare: 0.0034305783841 degrees of freedom: 1

Salad dressing, italian dressing, reduced fat 2

Salad dressing, french dressing, commercial, regular 1

Beverages 4 localChiSquare: 0.00228339004268 degrees of freedom: 1

Coffee, instant, decaffeinated, powder 1

Fruits and Fruit Juices

--overall chi-square: 0.446360692199 degrees of freedom: 8.0

Vegetables and Vegetable Products 9127 localChiSquare: 0.235603058596 degrees of freedom: 1

Squash, zucchini, baby, raw 94

Peas and carrots, frozen, cooked, boiled, drained, without salt 92

Lambsquarters, cooked, boiled, drained, without salt 86

Lambs quarters, cooked, boiled, drained, with salt 86

Cauliflower, cooked, boiled, drained, with salt 80

Squash, summer, zucchini, includes skin, frozen, cooked, boiled, drained, with salt 80

Cauliflower, green, cooked, no salt added 80

Cauliflower, green, cooked, with salt 80

Legumes and Legume Products 2912 localChiSquare: 0.0403438257158 degrees of freedom: 1

Soy flour, defatted 70

Soy meal, defatted, raw 70

Soy flour, low-fat, crude protein basis (N x 6.25) 70

Soy meal, defatted, raw, crude protein basis (N x 6.25) 70

Soybeans, mature seeds, cooked, boiled, with salt 69

Soy flour, defatted, crude protein basis (N x 6.25) 69

Soy flour, full-fat, raw 68

Soy flour, full-fat, roasted 68

MORI-NU, Tofu, silken, firm 68

Nut and Seed Products 472 localChiSquare: 0.0457858010605 degrees of freedom: 1

Seeds, pumpkin and squash seeds, whole, roasted, without salt 95

Seeds, lotus seeds, dried 62

Seeds, lotus seeds, raw 62

Seeds, sisymbrium sp. seeds, whole, dried 19

Seeds, cottonseed flour, low fat (glandless) 12

Seeds, cottonseed meal, partially defatted (glandless) 11

Nuts, formulated, wheat-based, flavored, macadamia flavored, without salt 11

Spices and Herbs 343 localChiSquare: 0.00771386781449 degrees of freedom: 1

Spices, fenugreek seed 72

Spices, parsley, dried 64

Spices, mustard seed, ground 49

Fruits and Fruit Juices 164 localChiSquare: 0.0907380361535 degrees of freedom: 1

Avocados, raw, Florida 31

Avocados, raw, all commercial varieties 13

Avocados, raw, California 9

Raisins, seedless 8

Melons, cantaloupe, raw 6

Applesauce, canned, sweetened, without salt (includes USDA commodity) 4

Applesauce, canned, sweetened, with salt 4

Persimmons, japanese, raw 4

Cereal Grains and Pasta 128 localChiSquare: 0.117945737403 degrees of freedom: 1

Wheat germ, crude 47

Amaranth, uncooked 19

Quinoa, uncooked 14

Quinoa, cooked 13

Tapioca, pearl, dry 8

Buckwheat 4

Buckwheat groats, roasted, cooked 4

Buckwheat flour, whole-groat 4

Arrowroot flour 3

Breakfast Cereals 84 localChiSquare: 0.0447557378181 degrees of freedom: 1

Cereals ready-to-eat, wheat germ, toasted, plain 49

Cereals, oats, regular and quick and instant, unenriched, cooked with water (includes boiling and microwaving), without salt 10

Cereals, oats, instant, fortified, plain, dry 8

Incaparina, dry mix (corn and soy flours), unprepared 7

Cereals, oats, instant, fortified, plain, prepared with water (boiling water added or microwaved) 6

Cereals, oats, regular and quick and instant, not fortified, dry 2

Cereals, farina, enriched, cooked with water, with salt 1

Fats and Oils 14 localChiSquare: 0.00292181342195 degrees of freedom: 1

Salad dressing, french dressing, reduced fat 8

Margarine, regular, hard, soybean (hydrogenated) 3

Beverages 1 localChiSquare: 0.00305286941483 degrees of freedom: 1

Orange drink, breakfast type, with juice and pulp, frozen concentrate 1

Vegetables and Vegetable Products

--overall chi-square: 0.0888049352608 degrees of freedom: 8.0

Vegetables and Vegetable Products 73648 localChiSquare: 0.0433009966352 degrees of freedom: 1

Squash, zucchini, baby, raw 618

Lambsquarters, cooked, boiled, drained, without salt 614

Lambs quarters, cooked, boiled, drained, with salt 614

Lambsquarters, raw 581

Squash, summer, zucchini, includes skin, frozen, cooked, boiled, drained, with salt 500

Asparagus, frozen, cooked, boiled, drained, with salt 447

Squash, summer, all varieties, raw 434

Squash, summer, zucchini, includes skin, raw 428

Legumes and Legume Products 23940 localChiSquare: 0.00747006934125 degrees of freedom: 1

MORI-NU, Tofu, silken, soft 416

Soy sauce made from soy and wheat (shoyu), low sodium 359

Tofu, extra firm, prepared with nigari 315

Tofu, soft, prepared with calcium sulfate and magnesium chloride (nigari) 312

Tofu, raw, firm, prepared with calcium sulfate 312

Tofu, raw, regular, prepared with calcium sulfate 310

Tofu, dried-frozen (koyadofu), prepared with calcium sulfate 303

Cereal Grains and Pasta 9639 localChiSquare: 0.0232901560145 degrees of freedom: 1

Wheat germ, crude 362

Wheat bran, crude 221

Macaroni, protein-fortified, dry, enriched, (n x 6.25) 131

Spaghetti, protein-fortified, dry, enriched (n x 6.25) 130

Spaghetti, protein-fortified, dry, enriched (n x 5.70) 124

Macaroni, protein-fortified, dry, enriched, (n x 5.70) 122

Macaroni, protein-fortified, cooked, enriched, (n x 5.70) 108

Macaroni, protein-fortified, cooked, enriched, (n x 6.25) 108

Nut and Seed Products 9531 localChiSquare: 0.00899875952089 degrees of freedom: 1

Seeds, cottonseed meal, partially defatted (glandless) 349

Seeds, sunflower seed flour, partially defatted 303

Seeds, pumpkin and squash seeds, whole, roasted, without salt 301

Seeds, pumpkin and squash seeds, whole, roasted, with salt added 301

Seeds, cottonseed kernels, roasted (glandless) 278

Seeds, sesame flour, low-fat 258

Seeds, safflower seed meal, partially defatted 241

Seeds, sesame flour, partially defatted 231

Fruits and Fruit Juices 9127 localChiSquare: 0.0140288891701 degrees of freedom: 1

Loquats, raw 93

Bananas, raw 92

Apricots, dried, sulfured, stewed, without added sugar 91

Apricots, dried, sulfured, stewed, with added sugar 91

Plums, dried (prunes), uncooked 91

Strawberries, canned, heavy syrup pack, solids and liquids 91

Strawberries, frozen, sweetened, whole 91

Breakfast Cereals 3928 localChiSquare: 0.0120526785043 degrees of freedom: 1

Cereals ready-to-eat, wheat germ, toasted, plain 338

Cereals, CREAM OF WHEAT, 1 minute cook time, cooked with water, microwaved, without salt 117

Cereals, oats, regular and quick and instant, unenriched, cooked with water (includes boiling and microwaving), without salt 85

Incaparina, dry mix (corn and soy flours), unprepared 74

Cereals, oats, instant, fortified, plain, prepared with water (boiling water added or microwaved) 64

Cereals, corn grits, white, regular and quick, enriched, cooked with water, without salt 56

Cereals, farina, enriched, cooked with water, without salt 56

Cereals, corn grits, white, regular and quick, enriched, cooked with water, with salt 56

Spices and Herbs 3154 localChiSquare: 0.0047719245423 degrees of freedom: 1

Mustard, prepared, yellow 335

Basil, fresh 333

Spices, parsley, dried 283

Spices, caraway seed 283

Spices, fenugreek seed 237

Spearmint, dried 177

Dill weed, fresh 100

Spices, ginger, ground 90

Fats and Oils 443 localChiSquare: 0.00045528371931 degrees of freedom: 1

Margarine-like, vegetable oil spread, unspecified oils, approximately 37 fat, with salt 91

Margarine, regular, hard, soybean (hydrogenated) 89

Salad dressing, french dressing, reduced fat 80

Salad dressing, french dressing, commercial, regular 73

Sandwich spread, with chopped pickle, regular, unspecified oils 64

Beverages 204 localChiSquare: 0.000871758613298 degrees of freedom: 1

Orange drink, breakfast type, with juice and pulp, frozen concentrate 53

Coffee, instant, regular, powder 51

Coffee, instant, decaffeinated, powder 50

Nut and Seed Products

--overall chi-square: 0.648612477208 degrees of freedom: 8.0

Vegetables and Vegetable Products 9531 localChiSquare: 0.0235449558527 degrees of freedom: 1

Lambsquarters, raw 131

Lambsquarters, cooked, boiled, drained, without salt 126

Lambs quarters, cooked, boiled, drained, with salt 126

Cauliflower, green, cooked, no salt added 113

Cauliflower, green, cooked, with salt 113

Cauliflower, green, raw 109

Asparagus, frozen, cooked, boiled, drained, with salt 104

Legumes and Legume Products 6987 localChiSquare: 0.439524611196 degrees of freedom: 1

MORI-NU, Tofu, silken, lite extra firm 124

Soy protein concentrate, produced by acid wash 124

Soy protein concentrate, crude protein basis (N x 6.25), produced by acid wash 124

MOR-NU, Tofu, silken, lite firm 123

Soy meal, defatted, raw, crude protein basis (N x 6.25) 122

Tofu, raw, firm, prepared with calcium sulfate 122

Soy protein isolate, PROTEIN TECHNOLOGIES INTERNATIONAL, SUPRO 121

Soy flour, defatted, crude protein basis (N x 6.25) 121

Fruits and Fruit Juices 472 localChiSquare: 0.0659872358516 degrees of freedom: 1

Pineapple, frozen, chunks, sweetened 11

Watermelon, raw 11

Pineapple, canned, extra heavy syrup pack, solids and liquids 9

Lime juice, raw 8

Pineapple, canned, heavy syrup pack, solids and liquids 8

Applesauce, canned, unsweetened, with added ascorbic acid 8

Apricots, raw 7

Apricots, canned, water pack, without skin, solids and liquids 7

Nut and Seed Products 462 localChiSquare: 0.0615676498753 degrees of freedom: 1

Seeds, lotus seeds, dried 52

Seeds, pumpkin and squash seeds, whole, roasted, without salt 50

Seeds, lotus seeds, raw 49

Seeds, breadfruit seeds, roasted 12

Seeds, cottonseed flour, partially defatted (glandless) 7

Seeds, cottonseed flour, low fat (glandless) 7

Seeds, cottonseed meal, partially defatted (glandless) 7

Seeds, safflower seed meal, partially defatted 7

Seeds, sesame flour, partially defatted 7

Seeds, sesame flour, low-fat 7

Spices and Herbs 358 localChiSquare: 0.00136789468893 degrees of freedom: 1

Spices, parsley, dried 115

Spices, fenugreek seed 114

Mustard, prepared, yellow 18

Spices, garlic powder 18

Dill weed, fresh 16

Spearmint, dried 7

Spices, oregano, dried 6

Basil, fresh 6

Cereal Grains and Pasta 188 localChiSquare: 0.116539214111 degrees of freedom: 1

Wheat germ, crude 95

Oat bran, raw 7

Wheat bran, crude 7

Cornstarch 5

Oat bran, cooked 5

Amaranth, uncooked 4

Arrowroot flour 4

Buckwheat groats, roasted, dry 4

Buckwheat groats, roasted, cooked 4

Rice, brown, medium-grain, raw 4

Breakfast Cereals 162 localChiSquare: 0.0400935231821 degrees of freedom: 1

Cereals ready-to-eat, wheat germ, toasted, plain 98

Incaparina, dry mix (corn and soy flours), unprepared 9

Cereals ready-to-eat, KELLOGG, KELLOGG'S APPLE JACKS 4

Fats and Oils 22 localChiSquare: 0.00268774546906 degrees of freedom: 1

Salad dressing, french dressing, reduced fat 6

Sandwich spread, with chopped pickle, regular, unspecified oils 4

Beverages 6 localChiSquare: 0.00257487231537 degrees of freedom: 1

Orange drink, breakfast type, with juice and pulp, frozen concentrate 4

Coffee, instant, with chicory, powder 2

Beverages

--overall chi-square: 0.875225270629 degrees of freedom: 8.0

Vegetables and Vegetable Products 204 localChiSquare: 0.0144318934422 degrees of freedom: 1

Horseradish-tree leafy tips, raw 6

Cauliflower, cooked, boiled, drained, with salt 4

Cowpeas (Blackeyes), immature seeds, frozen, cooked, boiled, drained, with salt 4

Horseradish-tree, leafy tips, cooked, boiled, drained, with salt 4

Cauliflower, cooked, boiled, drained, without salt 4

Cauliflower, frozen, unprepared 4

Cornsalad, raw 4

Cowpeas (Blackeyes), immature seeds, frozen, unprepared 4

Cowpeas (blackeyes), immature seeds, frozen, cooked, boiled, drained, without salt 4

Yardlong bean, raw 4

Legumes and Legume Products 174 localChiSquare: 0.639822917002 degrees of freedom: 1

Tofu, raw, regular, prepared with calcium sulfate 5

Soy protein isolate 4

Spices and Herbs 9 localChiSquare: 0.00349496832723 degrees of freedom: 1

Spices, fenugreek seed 4

Spices, parsley, dried 4

Spices, cinnamon, ground 1

Nut and Seed Products 6 localChiSquare: 0.080137394956 degrees of freedom: 1

Seeds, lotus seeds, raw 2

Seeds, lotus seeds, dried 2

Seeds, pumpkin and squash seeds, whole, roasted, without salt 1

Breakfast Cereals 4 localChiSquare: 0.0382705460164 degrees of freedom: 1

Cereals ready-to-eat, wheat germ, toasted, plain 4

Cereal Grains and Pasta 4 localChiSquare: 0.117346037495 degrees of freedom: 1

Wheat germ, crude 4

Fruits and Fruit Juices 1 localChiSquare: 0.111265891884 degrees of freedom: 1

Apples, frozen, unsweetened, heated 1

Legumes and Legume Products

--overall chi-square: 0.106727725064 degrees of freedom: 8.0

Vegetables and Vegetable Products 23940 localChiSquare: 0.00170446239611 degrees of freedom: 1

Mustard greens, cooked, boiled, drained, without salt 222

Mustard greens, cooked, boiled, drained, with salt 222

Asparagus, frozen, cooked, boiled, drained, with salt 219

Carrots, canned, no salt added, solids and liquids 208

Carrots, canned, regular pack, drained solids 207

Lambsquarters, raw 207

Mustard greens, frozen, unprepared 206

Cereal Grains and Pasta 8814 localChiSquare: 0.0235935200729 degrees of freedom: 1

Macaroni, protein-fortified, dry, enriched, (n x 6.25) 152

Spaghetti, protein-fortified, dry, enriched (n x 6.25) 151

Macaroni, protein-fortified, dry, enriched, (n x 5.70) 143

Spaghetti, protein-fortified, dry, enriched (n x 5.70) 141

Spaghetti, protein-fortified, cooked, enriched (n x 6.25) 104

Macaroni, protein-fortified, cooked, enriched, (n x 5.70) 103

Macaroni, protein-fortified, cooked, enriched, (n x 6.25) 103

Nut and Seed Products 6987 localChiSquare: 0.0151627463784 degrees of freedom: 1

Seeds, cottonseed kernels, roasted (glandless) 201

Seeds, sesame flour, partially defatted 197

Seeds, sesame flour, low-fat 194

Seeds, sunflower seed flour, partially defatted 194

Seeds, sesame flour, high-fat 194

Seeds, watermelon seed kernels, dried 167

Legumes and Legume Products 3622 localChiSquare: 0.0476363812444 degrees of freedom: 1

MORI-NU, Tofu, silken, soft 203

Peanut flour, defatted 194

Soy sauce made from soy and wheat (shoyu) 194

Soy sauce made from soy and wheat (shoyu), low sodium 194

Peanut flour, low fat 110

Peanuts, virginia, raw 45

Peanuts, spanish, oil-roasted, with salt 44

Peanuts, valencia, raw 44

Peanuts, valencia, oil-roasted, with salt 44

Peanuts, virginia, oil-roasted, with salt 44

Breakfast Cereals 3358 localChiSquare: 0.00253706146407 degrees of freedom: 1

Cereals, oats, regular and quick and instant, unenriched, cooked with water (includes boiling and microwaving), without salt 98

Cereals, oats, instant, fortified, plain, prepared with water (boiling water added or microwaved) 60

Cereals, oats, instant, fortified, plain, dry 57

Cereals, oats, regular and quick and instant, not fortified, dry 55

Cereals, farina, unenriched, dry 48

Cereals ready-to-eat, wheat, shredded, plain, sugar and salt free 48

Cereals, CREAM OF WHEAT, regular, 10 minute cooking, dry 47

Cereals, CREAM OF WHEAT, regular (10 minute), cooked with water, without salt 47

Cereals, CREAM OF WHEAT, instant, dry 47

Cereals, CREAM OF WHEAT, mix'n eat, plain, dry 47

Fruits and Fruit Juices 2912 localChiSquare: 0.0241936581935 degrees of freedom: 1

Peaches, canned, water pack, solids and liquids 47

Pineapple, raw, all varieties 47

Tangerine juice, frozen concentrate, sweetened, diluted with 3 volume water 46

Oranges, raw, California, valencias 45

Oranges, raw, Florida 45

Tangerines, (mandarin oranges), canned, juice pack 45

Pineapple, canned, water pack, solids and liquids 45

Spices and Herbs 1222 localChiSquare: 0.00511293121547 degrees of freedom: 1

Spices, caraway seed 179

Basil, fresh 174

Mustard, prepared, yellow 165

Spices, poppy seed 55

Spices, cinnamon, ground 51

Spices, fennel seed 49

Spices, garlic powder 44

Spearmint, dried 42

Beverages 174 localChiSquare: 1.39932574086e-05 degrees of freedom: 1

Coffee, instant, decaffeinated, powder 47

Fats and Oils 97 localChiSquare: 0.00175408638116 degrees of freedom: 1

Salad dressing, italian dressing, reduced fat 45

Sandwich spread, with chopped pickle, regular, unspecified oils 28

Salad dressing, french dressing, commercial, regular 20

Cereal Grains and Pasta

--overall chi-square: 0.921011345839 degrees of freedom: 8.0

Vegetables and Vegetable Products 9639 localChiSquare: 0.00735965936298 degrees of freedom: 1

Horseradish-tree, leafy tips, cooked, boiled, drained, with salt 189

Cauliflower, green, raw 186

Cauliflower, green, cooked, no salt added 183

Cauliflower, green, cooked, with salt 183

Jute, potherb, cooked, boiled, drained, with salt 174

Legumes and Legume Products 8814 localChiSquare: 0.711831861241 degrees of freedom: 1

Soy protein isolate, PROTEIN TECHNOLOGIES INTERNATIONAL, SUPRO 191

Soy protein isolate, potassium type, crude protein basis 190

Soy protein concentrate, crude protein basis (N x 6.25), produced by acid wash 189

Soy protein isolate, potassium type 189

Soy protein concentrate, produced by acid wash 188

MORI-NU, Tofu, silken, extra firm 186

MORI-NU, Tofu, silken, lite extra firm 186

Soy protein isolate, PROTEIN TECHNOLOGIES INTERNATIONAL, ProPlus 186

Spices and Herbs 375 localChiSquare: 0.00102611048988 degrees of freedom: 1

Spices, parsley, dried 177

Spices, fenugreek seed 173

Spices, mustard seed, ground 5

Spices, cinnamon, ground 4

Dill weed, fresh 3

Mustard, prepared, yellow 3

Rosemary, fresh 1

Cereal Grains and Pasta 280 localChiSquare: 0.108531146945 degrees of freedom: 1

Wheat germ, crude 140

Amaranth, uncooked 1

Breakfast Cereals 221 localChiSquare: 0.0360698403758 degrees of freedom: 1

Cereals ready-to-eat, wheat germ, toasted, plain 150

Incaparina, dry mix (corn and soy flours), unprepared 6

Cereals, CREAM OF WHEAT, instant, prepared with water, with salt, (wheat) 1

Nut and Seed Products 188 localChiSquare: 0.0905854011069 degrees of freedom: 1

Seeds, lotus seeds, raw 36

Seeds, lotus seeds, dried 21

Seeds, pumpkin and squash seeds, whole, roasted, without salt 12

Seeds, pumpkin and squash seeds, whole, roasted, with salt added 11

Seeds, breadfruit seeds, raw 6

Fruits and Fruit Juices 128 localChiSquare: 0.102664119355 degrees of freedom: 1

Applesauce, canned, unsweetened, with added ascorbic acid 8

Pineapple, frozen, chunks, sweetened 6

Melons, cantaloupe, raw 3

Pineapple, canned, heavy syrup pack, solids and liquids 3

Pineapple, canned, extra heavy syrup pack, solids and liquids 3

Oranges, raw, navels 3

Fats and Oils 7 localChiSquare: 0.00412248275321 degrees of freedom: 1

Salad dressing, french dressing, reduced fat 2

Sandwich spread, with chopped pickle, regular, unspecified oils 1

Beverages 4 localChiSquare: 0.0028072721568 degrees of freedom: 1

Coffee, instant, decaffeinated, powder 1
