# Supplementary material for: vProtein: Identifying Optimal Amino Acid Complements from Plant-Based Foods
Source: PLoS One. 2011 Apr 22;6(4):e18836. doi: 10.1371/journal.pone.0018836 (PMC3081312; doi:10.1371/journal.pone.0018836)
Supplement: Table S3 — Minimum Weight: Overall food group based pairings, chi-square statistics, and top-food matches. (DOCX) [file pone.0018836.s006.docx]

**Table S3.** Minimum Weight: Overall food group based pairings, chi-square statistics, and top-food matches.

Spices and Herbs

--overall chi-square: 0.0558311364347 degrees of freedom: 8.0

Vegetables and Vegetable Products 9629 localChiSquare: 0.0041097666038 degrees of freedom: 1

Edamame, frozen, unprepared 44

Edamame, frozen, prepared 42

Asparagus, frozen, cooked, boiled, drained, with salt 39

Squash, zucchini, baby, raw 37

Lambsquarters, raw 36

Asparagus, canned, no salt added, solids and liquids 35

Legumes and Legume Products 4201 localChiSquare: 0.0292443225598 degrees of freedom: 1

Winged beans, mature seeds, raw 40

Soy sauce made from soy (tamari) 39

Meat extender 37

Beans, kidney, royal red, mature seeds, raw 36

Nut and Seed Products 1808 localChiSquare: 0.00104924794053 degrees of freedom: 1

Seeds, pumpkin and squash seeds, whole, roasted, without salt 32

Seeds, pumpkin and squash seeds, whole, roasted, with salt added 32

Nuts, cashew nuts, oil roasted, without salt added 25

Nuts, cashew nuts, oil roasted, with salt added 25

Seeds, sunflower seed flour, partially defatted 24

Nuts, cashew nuts, raw 23

Cereal Grains and Pasta 1741 localChiSquare: 0.0116841950343 degrees of freedom: 1

Wheat germ, crude 44

Buckwheat groats, roasted, dry 19

Oat bran, raw 19

Quinoa, uncooked 19

Rice bran, crude 19

Buckwheat flour, whole-groat 18

Buckwheat 17

Wheat bran, crude 17

Amaranth, uncooked 16

Fruits and Fruit Juices 1428 localChiSquare: 0.0119323700848 degrees of freedom: 1

Cranberries, raw 19

Apricots, dehydrated (low-moisture), sulfured, stewed 17

Applesauce, canned, unsweetened, with added ascorbic acid 17

Apricots, dehydrated (low-moisture), sulfured, uncooked 16

Orange juice, chilled, includes from concentrate 16

Orange juice, chilled, includes from concentrate, fortified with calcium and vitamin D 16

Orange juice, chilled, includes from concentrate, fortified with calcium 16

Breakfast Cereals 776 localChiSquare: 0.00475287991985 degrees of freedom: 1

Cereals ready-to-eat, wheat germ, toasted, plain 47

Incaparina, dry mix (corn and soy flours), unprepared 21

Cereals, oats, regular and quick and instant, not fortified, dry 15

Cereals, corn grits, white, regular, quick, unenriched, cooked with water, with salt 14

Cereals, corn grits, yellow, regular and quick, enriched, cooked with water, without salt 14

Cereals, corn grits, yellow, regular, quick, enriched, cooked with water, with salt 14

Cereals ready-to-eat, KELLOGG, KELLOGG'S CRISPIX 13

Cereals, corn grits, white, regular and quick, unenriched, dry 13

Cereals, corn grits, yellow, regular and quick, enriched, dry 13

Spices and Herbs 390 localChiSquare: 0.00121612968029 degrees of freedom: 1

Spices, parsley, dried 38

Spices, fenugreek seed 32

Spices, garlic powder 28

Spices, poppy seed 21

Spices, cinnamon, ground 21

Mustard, prepared, yellow 21

Spices, caraway seed 19

Fats and Oils 71 localChiSquare: 0.000328986586397 degrees of freedom: 1

Salad dressing, french dressing, commercial, regular 14

Margarine-like, vegetable oil spread, unspecified oils, approximately 37 fat, with salt 13

Sandwich spread, with chopped pickle, regular, unspecified oils 12

Beverages 33 localChiSquare: 0.000753694826255 degrees of freedom: 1

Orange drink, breakfast type, with juice and pulp, frozen concentrate 9

Coffee, instant, decaffeinated, powder 8

Fats and Oils

--overall chi-square: 0.235767792777 degrees of freedom: 8.0

Vegetables and Vegetable Products 1440 localChiSquare: 0.127483685488 degrees of freedom: 1

Peas and carrots, frozen, cooked, boiled, drained, without salt 21

Edamame, frozen, prepared 12

Lambsquarters, raw 12

Lambsquarters, cooked, boiled, drained, without salt 11

Lambs quarters, cooked, boiled, drained, with salt 11

Squash, zucchini, baby, raw 11

Edamame, frozen, unprepared 10

Tomatoes, sun-dried 10

Legumes and Legume Products 350 localChiSquare: 9.86855555711e-05 degrees of freedom: 1

Falafel, home-prepared 9

Hummus, home prepared 8

Bacon, meatless 6

Meat extender 6

Sausage, meatless 6

Soybeans, mature seeds, raw 6

Soybeans, mature cooked, boiled, without salt 6

Soybeans, mature seeds, roasted, salted 6

Soybeans, mature seeds, dry roasted 6

Soy flour, full-fat, raw 6

Fruits and Fruit Juices 247 localChiSquare: 7.80035616732e-05 degrees of freedom: 1

Avocados, raw, Florida 8

Avocados, raw, all commercial varieties 7

Avocados, raw, California 7

Pineapple, frozen, chunks, sweetened 6

Apricots, canned, juice pack, with skin, solids and liquids 4

Apricots, canned, heavy syrup pack, without skin, solids and liquids 4

Apricots, canned, extra heavy syrup pack, without skin, solids and liquids 4

Apricots, dried, sulfured, stewed, with added sugar 4

Oranges, raw, all commercial varieties 4

Oranges, raw, California, valencias 4

Nut and Seed Products 123 localChiSquare: 0.0239232157346 degrees of freedom: 1

Seeds, pumpkin and squash seeds, whole, roasted, without salt 12

Nuts, coconut meat, dried (desiccated), sweetened, flaked, packaged 6

Seeds, lotus seeds, raw 6

Nuts, beechnuts, dried 5

Nuts, chestnuts, european, dried, unpeeled 5

Nuts, chestnuts, european, dried, peeled 5

Nuts, formulated, wheat-based, flavored, macadamia flavored, without salt 5

Nuts, acorns, raw 4

Nuts, acorns, dried 4

Nuts, acorn flour, full fat 4

Spices and Herbs 71 localChiSquare: 0.0164337064412 degrees of freedom: 1

Spices, cinnamon, ground 13

Spices, garlic powder 13

Spices, mustard seed, ground 12

Cereal Grains and Pasta 43 localChiSquare: 0.0998064205036 degrees of freedom: 1

Wheat germ, crude 15

Breakfast Cereals 25 localChiSquare: 0.0367232760471 degrees of freedom: 1

Cereals ready-to-eat, wheat germ, toasted, plain 15

Incaparina, dry mix (corn and soy flours), unprepared 4

Cereals, CREAM OF WHEAT, 1 minute cook time, cooked with water, stove-top, without salt 2

Cereals, oats, instant, fortified, plain, dry 1

Fats and Oils 4 localChiSquare: 0.00195412248729 degrees of freedom: 1

Salad dressing, french dressing, reduced fat 2

Breakfast Cereals

--overall chi-square: 0.560187790705 degrees of freedom: 8.0

Vegetables and Vegetable Products 12022 localChiSquare: 8.44443302959e-05 degrees of freedom: 1

Asparagus, frozen, cooked, boiled, drained, with salt 171

Asparagus, canned, no salt added, solids and liquids 167

Horseradish-tree leafy tips, raw 149

Cauliflower, green, raw 146

Legumes and Legume Products 10233 localChiSquare: 0.442641616935 degrees of freedom: 1

Tofu, dried-frozen (koyadofu) 148

Tofu, dried-frozen (koyadofu), prepared with calcium sulfate 148

Soy flour, full-fat, roasted 147

Soy flour, full-fat, raw, crude protein basis (N x 6.25) 147

Soy flour, full-fat, roasted, crude protein basis (N x 6.25) 147

Winged beans, mature seeds, raw 146

Soybeans, mature seeds, roasted, no salt added 146

Fruits and Fruit Juices 1288 localChiSquare: 0.0336072962553 degrees of freedom: 1

Pineapple, frozen, chunks, sweetened 82

Applesauce, canned, unsweetened, with added ascorbic acid 68

Watermelon, raw 55

Applesauce, canned, sweetened, with salt 52

Applesauce, canned, sweetened, without salt (includes USDA commodity) 51

Nut and Seed Products 841 localChiSquare: 0.0516696373396 degrees of freedom: 1

Seeds, lotus seeds, dried 87

Seeds, pumpkin and squash seeds, whole, roasted, with salt added 69

Seeds, pumpkin and squash seeds, whole, roasted, without salt 68

Seeds, lotus seeds, raw 29

Nuts, coconut meat, dried (desiccated), sweetened, flaked, packaged 27

Seeds, breadnut tree seeds, dried 23

Nuts, beechnuts, dried 16

Nuts, chestnuts, european, dried, unpeeled 16

Seeds, breadfruit seeds, raw 10

Cereal Grains and Pasta 836 localChiSquare: 0.0766653111171 degrees of freedom: 1

Wheat germ, crude 201

Wild rice, raw 7

Wheat bran, crude 6

Macaroni, protein-fortified, dry, enriched, (n x 5.70) 6

Spaghetti, protein-fortified, dry, enriched (n x 5.70) 6

Macaroni, protein-fortified, dry, enriched, (n x 6.25) 6

Spaghetti, protein-fortified, dry, enriched (n x 6.25) 6

Semolina, enriched 5

Sorghum 5

Wheat, hard red spring 5

Spices and Herbs 776 localChiSquare: 0.0131834087633 degrees of freedom: 1

Spices, fenugreek seed 209

Spices, garlic powder 178

Spices, parsley, dried 145

Spices, cinnamon, ground 68

Spices, onion powder 46

Spices, oregano, dried 10

Dill weed, fresh 10

Spices, ginger, ground 6

Spices, poppy seed 5

Mustard, prepared, yellow 5

Breakfast Cereals 544 localChiSquare: 0.0222633977128 degrees of freedom: 1

Cereals ready-to-eat, wheat germ, toasted, plain 202

Incaparina, dry mix (corn and soy flours), unprepared 74

Cereals, oats, regular and quick and instant, not fortified, dry 6

Cereals, oats, regular and quick and instant, unenriched, cooked with water (includes boiling and microwaving), without salt 5

Cereals, oats, instant, fortified, plain, dry 5

Cereals, CREAM OF WHEAT, instant, prepared with water, with salt, (wheat) 4

Fats and Oils 25 localChiSquare: 0.0031070157362 degrees of freedom: 1

Salad dressing, italian dressing, reduced fat 5

Sandwich spread, with chopped pickle, regular, unspecified oils 4

Beverages 15 localChiSquare: 0.00217036039596 degrees of freedom: 1

Coffee, instant, decaffeinated, powder 4

Fruits and Fruit Juices

--overall chi-square: 0.166762843972 degrees of freedom: 8.0

Vegetables and Vegetable Products 27197 localChiSquare: 0.0114997722724 degrees of freedom: 1

Peas and carrots, frozen, cooked, boiled, drained, without salt 244

Cauliflower, green, cooked, with salt 232

Cauliflower, green, cooked, no salt added 230

Cauliflower, green, raw 224

Lambsquarters, cooked, boiled, drained, without salt 223

Lambs quarters, cooked, boiled, drained, with salt 223

Lambsquarters, raw 220

Squash, zucchini, baby, raw 200

Cauliflower, frozen, cooked, boiled, drained, with salt 199

Legumes and Legume Products 13433 localChiSquare: 0.077585156706 degrees of freedom: 1

Falafel, home-prepared 197

Soy flour, defatted 182

Soy flour, low-fat, crude protein basis (N x 6.25) 182

Soy flour, defatted, crude protein basis (N x 6.25) 181

Soy meal, defatted, raw 178

Winged beans, mature seeds, raw 178

Soy meal, defatted, raw, crude protein basis (N x 6.25) 178

Soy flour, full-fat, roasted 175

Soy flour, full-fat, roasted, crude protein basis (N x 6.25) 175

Nut and Seed Products 5017 localChiSquare: 0.000598657191734 degrees of freedom: 1

Seeds, pumpkin and squash seeds, whole, roasted, without salt 253

Seeds, pumpkin and squash seeds, whole, roasted, with salt added 253

Nuts, coconut meat, dried (desiccated), sweetened, flaked, packaged 157

Nuts, formulated, wheat-based, flavored, macadamia flavored, without salt 152

Seeds, lotus seeds, raw 140

Nuts, formulated, wheat-based, all flavors except macadamia, without salt 132

Cereal Grains and Pasta 2981 localChiSquare: 0.0421115775851 degrees of freedom: 1

Wheat germ, crude 200

Amaranth, uncooked 130

Fruits and Fruit Juices 2616 localChiSquare: 0.0339231159626 degrees of freedom: 1

Applesauce, canned, unsweetened, with added ascorbic acid 66

Dates, medjool 63

Pineapple, canned, juice pack, solids and liquids 55

Pineapple, frozen, chunks, sweetened 55

Spices and Herbs 1428 localChiSquare: 0.00831665563968 degrees of freedom: 1

Spices, fenugreek seed 229

Spices, garlic powder 143

Spices, cinnamon, ground 142

Spices, caraway seed 103

Mustard, prepared, yellow 68

Spices, poppy seed 56

Spices, basil, dried 46

Breakfast Cereals 1288 localChiSquare: 0.0181735450161 degrees of freedom: 1

Incaparina, dry mix (corn and soy flours), unprepared 71

Cereals, oats, instant, fortified, plain, dry 54

Cereals ready-to-eat, wheat, shredded, plain, sugar and salt free 40

Cereals, oats, regular and quick and instant, not fortified, dry 39

Cereals, oats, regular and quick and instant, unenriched, cooked with water (includes boiling and microwaving), without salt 28

Cereals ready-to-eat, KELLOGG, KELLOGG'S ALL-BRAN Original 27

Cereals, oats, instant, fortified, plain, prepared with water (boiling water added or microwaved) 25

Fats and Oils 247 localChiSquare: 1.1888927106e-05 degrees of freedom: 1

Salad dressing, italian dressing, reduced fat 71

Margarine-like, vegetable oil spread, unspecified oils, approximately 37 fat, with salt 64

Margarine, regular, hard, soybean (hydrogenated) 62

Salad dressing, french dressing, reduced fat 28

Sandwich spread, with chopped pickle, regular, unspecified oils 19

Beverages 73 localChiSquare: 0.00107258023794 degrees of freedom: 1

Coffee, instant, decaffeinated, powder 23

Vegetables and Vegetable Products

--overall chi-square: 0.0503725610508 degrees of freedom: 8.0

Vegetables and Vegetable Products 176816 localChiSquare: 0.012212641063 degrees of freedom: 1

Squash, zucchini, baby, raw 1325

Lambs quarters, cooked, boiled, drained, with salt 1207

Squash, summer, zucchini, includes skin, frozen, cooked, boiled, drained, with salt 1010

Squash, summer, zucchini, includes skin, raw 967

Squash, summer, zucchini, includes skin, frozen, unprepared 952

Squash, summer, all varieties, raw 942

Legumes and Legume Products 59961 localChiSquare: 0.00383654577474 degrees of freedom: 1

Soy sauce made from soy and wheat (shoyu) 713

MORI-NU, Tofu, silken, soft 698

Soy sauce made from soy and wheat (shoyu), low sodium 692

Soy flour, low-fat, crude protein basis (N x 6.25) 617

Soy flour, defatted, crude protein basis (N x 6.25) 614

Soy meal, defatted, raw 609

Soy meal, defatted, raw, crude protein basis (N x 6.25) 609

Nut and Seed Products 36235 localChiSquare: 0.000117866030907 degrees of freedom: 1

Seeds, pumpkin and squash seeds, whole, roasted, with salt added 1038

Seeds, pumpkin and squash seeds, whole, roasted, without salt 1037

Nuts, coconut meat, dried (desiccated), sweetened, flaked, packaged 854

Seeds, cottonseed kernels, roasted (glandless) 602

Nuts, acorn flour, full fat 540

Cereal Grains and Pasta 27848 localChiSquare: 0.0171978398092 degrees of freedom: 1

Wheat germ, crude 1100

Amaranth, uncooked 590

Fruits and Fruit Juices 27197 localChiSquare: 0.00791987057796 degrees of freedom: 1

Pineapple, frozen, chunks, sweetened 415

Pineapple, canned, juice pack, solids and liquids 383

Peaches, raw 355

Pineapple, canned, water pack, solids and liquids 339

Cherries, sweet, raw 330

Pineapple, canned, light syrup pack, solids and liquids 305

Pineapple, canned, extra heavy syrup pack, solids and liquids 302

Pineapple, canned, heavy syrup pack, solids and liquids 298

Breakfast Cereals 12022 localChiSquare: 0.00786530964635 degrees of freedom: 1

Cereals ready-to-eat, wheat germ, toasted, plain 1036

Incaparina, dry mix (corn and soy flours), unprepared 359

Cereals, oats, instant, fortified, plain, dry 344

Cereals, oats, regular and quick and instant, not fortified, dry 291

Cereals, oats, instant, fortified, plain, prepared with water (boiling water added or microwaved) 227

Cereals, oats, regular and quick and instant, unenriched, cooked with water (includes boiling and microwaving), without salt 209

Cereals, farina, enriched, cooked with water, without salt 192

Spices and Herbs 9629 localChiSquare: 0.0099848891665 degrees of freedom: 1

Mustard, prepared, yellow 861

Spices, garlic powder 772

Spices, caraway seed 672

Spices, fenugreek seed 621

Spices, cinnamon, ground 614

Spearmint, dried 496

Basil, fresh 449

Spices, fennel seed 403

Fats and Oils 1440 localChiSquare: 0.000101260161822 degrees of freedom: 1

Margarine-like, vegetable oil spread, unspecified oils, approximately 37 fat, with salt 283

Sandwich spread, with chopped pickle, regular, unspecified oils 254

Beverages 600 localChiSquare: 0.000694466768404 degrees of freedom: 1

Orange drink, breakfast type, with juice and pulp, frozen concentrate 201

Coffee, instant, regular, powder 134

Coffee, instant, decaffeinated, powder 133

Nut and Seed Products

--overall chi-square: 0.56108810526 degrees of freedom: 8.0

Vegetables and Vegetable Products 36235 localChiSquare: 0.00217817366891 degrees of freedom: 1

Edamame, frozen, prepared 375

Lambsquarters, raw 370

Lambsquarters, cooked, boiled, drained, without salt 354

Lambs quarters, cooked, boiled, drained, with salt 354

Soybeans, mature seeds, sprouted, raw 254

Cauliflower, green, cooked, no salt added 254

Cauliflower, green, cooked, with salt 254

Asparagus, frozen, cooked, boiled, drained, with salt 251

Legumes and Legume Products 29244 localChiSquare: 0.42443749225 degrees of freedom: 1

Soy sauce made from soy (tamari) 344

Tofu, raw, firm, prepared with calcium sulfate 311

Falafel, home-prepared 310

Tofu, hard, prepared with nigari 300

Tofu, dried-frozen (koyadofu) 290

Tofu, dried-frozen (koyadofu), prepared with calcium sulfate 290

Winged beans, mature seeds, raw 288

Soybeans, mature seeds, cooked, boiled, with salt 286

Fruits and Fruit Juices 5017 localChiSquare: 0.0165354655915 degrees of freedom: 1

Apples, frozen, unsweetened, heated 152

Applesauce, canned, unsweetened, without added ascorbic acid (includes USDA commodity) 145

Applesauce, canned, unsweetened, with added ascorbic acid 145

Applesauce, canned, sweetened, without salt (includes USDA commodity) 122

Applesauce, canned, sweetened, with salt 122

Watermelon, raw 111

Nut and Seed Products 2562 localChiSquare: 0.0491864531263 degrees of freedom: 1

Seeds, pumpkin and squash seeds, whole, roasted, without salt 206

Seeds, pumpkin and squash seeds, whole, roasted, with salt added 206

Seeds, lotus seeds, dried 203

Seeds, lotus seeds, raw 98

Seeds, breadfruit seeds, raw 83

Spices and Herbs 1808 localChiSquare: 0.00465127843202 degrees of freedom: 1

Spices, fenugreek seed 387

Spices, garlic powder 276

Dill weed, fresh 136

Spices, cinnamon, ground 112

Spices, ginger, ground 65

Mustard, prepared, yellow 55

Cereal Grains and Pasta 1099 localChiSquare: 0.108454112772 degrees of freedom: 1

Wheat germ, crude 310

Cornstarch 18

Oat bran, raw 16

Rice bran, crude 16

Wheat bran, crude 16

Amaranth, uncooked 14

Breakfast Cereals 841 localChiSquare: 0.0365969039178 degrees of freedom: 1

Cereals ready-to-eat, wheat germ, toasted, plain 319

Incaparina, dry mix (corn and soy flours), unprepared 163

Cereals, CREAM OF RICE, cooked with water, without salt 11

Cereals, oats, instant, fortified, plain, dry 11

Cereals, CREAM OF RICE, cooked with water, with salt 11

Cereals ready-to-eat, GENERAL MILLS, CINNAMON TOAST CRUNCH 8

Cereals, oats, regular and quick and instant, not fortified, dry 8

Cereals, oats, instant, fortified, plain, prepared with water (boiling water added or microwaved) 8

Cereals, corn grits, white, regular, quick, unenriched, cooked with water, with salt 8

Cereals, corn grits, yellow, regular, quick, enriched, cooked with water, with salt 8

Fats and Oils 123 localChiSquare: 0.0021352290089 degrees of freedom: 1

Salad dressing, french dressing, reduced fat 31

Margarine, regular, hard, soybean (hydrogenated) 30

Margarine-like, vegetable oil spread, unspecified oils, approximately 37 fat, with salt 24

Salad dressing, french dressing, commercial, regular 14

Sandwich spread, with chopped pickle, regular, unspecified oils 12

Beverages 14 localChiSquare: 0.00284789480364 degrees of freedom: 1

Orange drink, breakfast type, with juice and pulp, frozen concentrate 9

Coffee, instant, with chicory, powder 3

Coffee, instant, decaffeinated, powder 1

Beverages

--overall chi-square: 0.758301001302 degrees of freedom: 8.0

Vegetables and Vegetable Products 600 localChiSquare: 0.00112395110839 degrees of freedom: 1

Horseradish-tree leafy tips, raw 10

Asparagus, frozen, unprepared 9

Asparagus, frozen, cooked, boiled, drained, without salt 9

Cauliflower, cooked, boiled, drained, with salt 9

Cauliflower, cooked, boiled, drained, without salt 9

Cauliflower, frozen, unprepared 9

Cauliflower, green, raw 9

Legumes and Legume Products 545 localChiSquare: 0.591244944022 degrees of freedom: 1

Falafel, home-prepared 10

Soy sauce made from soy (tamari) 9

Sausage, meatless 8

Soybeans, mature seeds, raw 8

Soybeans, mature cooked, boiled, without salt 8

Soybeans, mature seeds, roasted, salted 8

Soybeans, mature seeds, dry roasted 8

Soy flour, full-fat, raw 8

Soy flour, full-fat, roasted 8

Fruits and Fruit Juices 73 localChiSquare: 0.0246592690357 degrees of freedom: 1

Apples, frozen, unsweetened, heated 7

Apples, dried, sulfured, uncooked 6

Watermelon, raw 6

Apples, dehydrated (low moisture), sulfured, uncooked 5

Apples, canned, sweetened, sliced, drained, unheated 3

Apples, canned, sweetened, sliced, drained, heated 3

Apples, dehydrated (low moisture), sulfured, stewed 3

Apples, dried, sulfured, stewed, without added sugar 3

Apples, dried, sulfured, stewed, with added sugar 3

Apples, frozen, unsweetened, unheated 3

Spices and Herbs 33 localChiSquare: 0.00721234471431 degrees of freedom: 1

Spices, fenugreek seed 13

Spices, parsley, dried 8

Spices, garlic powder 6

Breakfast Cereals 15 localChiSquare: 0.0354579346183 degrees of freedom: 1

Cereals ready-to-eat, wheat germ, toasted, plain 12

Nut and Seed Products 14 localChiSquare: 0.0880496165159 degrees of freedom: 1

Seeds, lotus seeds, dried 6

Seeds, lotus seeds, raw 3

Seeds, pumpkin and squash seeds, whole, roasted, without salt 2

Seeds, pumpkin and squash seeds, whole, roasted, with salt added 2

Nuts, coconut meat, dried (desiccated), sweetened, flaked, packaged 1

Cereal Grains and Pasta 12 localChiSquare: 0.118736065459 degrees of freedom: 1

Wheat germ, crude 12

Legumes and Legume Products

--overall chi-square: 0.142209759106 degrees of freedom: 8.0

Vegetables and Vegetable Products 59961 localChiSquare: 0.0178181959746 degrees of freedom: 1

Carrots, canned, no salt added, solids and liquids 431

Tomatoes, sun-dried 431

Seaweed, spirulina, dried 429

Leeks, (bulb and lower-leaf portion), freeze-dried 422

Carrots, cooked, boiled, drained, with salt 421

Tomatoes, yellow, raw 378

Nut and Seed Products 29244 localChiSquare: 0.0827936654659 degrees of freedom: 1

Seeds, cottonseed kernels, roasted (glandless) 463

Seeds, sesame flour, high-fat 429

Nuts, chestnuts, japanese, dried 403

Cereal Grains and Pasta 25901 localChiSquare: 0.0169467679949 degrees of freedom: 1

Wheat germ, crude 734

Wheat bran, crude 426

Rice bran, crude 343

Macaroni, protein-fortified, dry, enriched, (n x 6.25) 314

Spaghetti, protein-fortified, dry, enriched (n x 6.25) 314

Macaroni, protein-fortified, dry, enriched, (n x 5.70) 306

Spaghetti, protein-fortified, dry, enriched (n x 5.70) 304

Fruits and Fruit Juices 13433 localChiSquare: 0.00390326259505 degrees of freedom: 1

Peaches, raw 338

Kiwifruit, gold, raw 323

Pineapple, raw, all varieties 312

Pineapple, canned, water pack, solids and liquids 307

Pineapple, canned, juice pack, solids and liquids 295

Nectarines, raw 277

Legumes and Legume Products 13366 localChiSquare: 0.0318350552769 degrees of freedom: 1

Peanut flour, defatted 434

Peanut flour, low fat 347

Peanuts, spanish, oil-roasted, without salt 283

Peanuts, valencia, oil-roasted, without salt 279

Soy sauce made from soy and wheat (shoyu) 275

Soy sauce made from soy and wheat (shoyu), low sodium 275

Peanuts, all types, oil-roasted, without salt 269

Breakfast Cereals 10233 localChiSquare: 0.00227747755713 degrees of freedom: 1

Cereals, oats, instant, fortified, plain, dry 405

Cereals, oats, regular and quick and instant, not fortified, dry 319

Cereals, oats, regular and quick and instant, unenriched, cooked with water (includes boiling and microwaving), without salt 226

Cereals, oats, instant, fortified, plain, prepared with water (boiling water added or microwaved) 181

Cereals ready-to-eat, wheat, puffed, fortified 166

Cereals, farina, unenriched, dry 165

Cereals ready-to-eat, wheat, shredded, plain, sugar and salt free 148

Spices and Herbs 4201 localChiSquare: 0.00894001275276 degrees of freedom: 1

Spices, caraway seed 428

Spices, garlic powder 392

Spices, cinnamon, ground 390

Mustard, prepared, yellow 327

Spices, poppy seed 318

Spices, fennel seed 243

Basil, fresh 233

Spearmint, dried 218

Spices, basil, dried 193

Beverages 545 localChiSquare: 2.35641317601e-05 degrees of freedom: 1

Coffee, instant, decaffeinated, powder 138

Fats and Oils 350 localChiSquare: 0.00137791038526 degrees of freedom: 1

Salad dressing, italian dressing, reduced fat 137

Sandwich spread, with chopped pickle, regular, unspecified oils 115

Cereal Grains and Pasta

--overall chi-square: 0.745456494688 degrees of freedom: 8.0

Vegetables and Vegetable Products 27848 localChiSquare: 0.000157798338203 degrees of freedom: 1

Lambsquarters, cooked, boiled, drained, without salt 353

Lambs quarters, cooked, boiled, drained, with salt 353

Cauliflower, green, raw 352

Cauliflower, green, cooked, no salt added 348

Cauliflower, green, cooked, with salt 348

Asparagus, canned, no salt added, solids and liquids 335

Cauliflower, cooked, boiled, drained, with salt 335

Legumes and Legume Products 25901 localChiSquare: 0.593001234165 degrees of freedom: 1

Soy sauce made from soy (tamari) 415

Soy protein isolate, PROTEIN TECHNOLOGIES INTERNATIONAL, SUPRO 383

Soy protein isolate, potassium type, crude protein basis 379

Soy protein concentrate, crude protein basis (N x 6.25), produced by acid wash 378

Soy protein isolate, potassium type 378

Soy protein concentrate, produced by acid wash 377

Soy protein isolate, PROTEIN TECHNOLOGIES INTERNATIONAL, ProPlus 376

Fruits and Fruit Juices 2981 localChiSquare: 0.0334419967902 degrees of freedom: 1

Pineapple, frozen, chunks, sweetened 181

Applesauce, canned, unsweetened, with added ascorbic acid 179

Applesauce, canned, sweetened, without salt (includes USDA commodity) 147

Applesauce, canned, sweetened, with salt 147

Watermelon, raw 141

Spices and Herbs 1741 localChiSquare: 0.0116990612687 degrees of freedom: 1

Spices, fenugreek seed 516

Spices, parsley, dried 359

Spices, garlic powder 344

Spices, mustard seed, ground 157

Spices, cinnamon, ground 154

Spices, onion powder 117

Dill weed, fresh 36

Mustard, prepared, yellow 19

Spices, ginger, ground 11

Spices, oregano, dried 8

Nut and Seed Products 1099 localChiSquare: 0.0745875748441 degrees of freedom: 1

Seeds, lotus seeds, dried 186

Seeds, pumpkin and squash seeds, whole, roasted, without salt 158

Seeds, pumpkin and squash seeds, whole, roasted, with salt added 157

Nuts, coconut meat, dried (desiccated), sweetened, flaked, packaged 89

Seeds, lotus seeds, raw 51

Seeds, breadnut tree seeds, dried 34

Seeds, breadfruit seeds, boiled 26

Seeds, breadfruit seeds, roasted 25

Seeds, breadfruit seeds, raw 23

Cereal Grains and Pasta 882 localChiSquare: 0.108263351232 degrees of freedom: 1

Wheat germ, crude 439

Rice, white, medium-grain, raw, enriched 4

Quinoa, cooked 4

Rice, white, medium-grain, raw, unenriched 4

Amaranth, uncooked 3

Breakfast Cereals 836 localChiSquare: 0.032182042497 degrees of freedom: 1

Cereals ready-to-eat, wheat germ, toasted, plain 455

Incaparina, dry mix (corn and soy flours), unprepared 184

Cereals, CREAM OF WHEAT, instant, prepared with water, with salt, (wheat) 3

Fats and Oils 43 localChiSquare: 0.00350595695183 degrees of freedom: 1

Salad dressing, french dressing, reduced fat 13

Margarine, regular, hard, soybean (hydrogenated) 11

Margarine-like, vegetable oil spread, unspecified oils, approximately 37 fat, with salt 10

Sandwich spread, with chopped pickle, regular, unspecified oils 3

Beverages 12 localChiSquare: 0.00282209213637 degrees of freedom: 1

Coffee, instant, decaffeinated, powder 3
